# Development of reciprocal connections between the dorsal lateral geniculate nucleus and the thalamic reticular nucleus

**DOI:** 10.1186/s13064-024-00183-5

**Published:** 2024-06-18

**Authors:** Peter W Campbell, Gubbi Govindaiah, William Guido

**Affiliations:** 1 Department of Anatomical Sciences and Neurobiology, University of Louisville School of Medicine, 511 S. Floyd St., Louisville, KY 40292 USA; 2 Division of Neurology and Developmental Neurosciences, Baylor College of Medicine, Houston, USA

**Keywords:** Dorsal lateral geniculate nucleus, Thalamic reticular nucleus, Development, Mouse

## Abstract

The thalamic reticular nucleus (TRN) serves as an important node between the thalamus and neocortex, regulating thalamocortical rhythms and sensory processing in a state dependent manner. Disruptions in TRN circuitry also figures prominently in several neurodevelopmental disorders including epilepsy, autism, and attentional defects. An understanding of how and when connections between TRN and 1st order thalamic nuclei, such as the dorsal lateral geniculate nucleus (dLGN), develop is lacking. We used the mouse visual thalamus as a model system to study the organization, pattern of innervation and functional responses between TRN and the dLGN. Genetically modified mouse lines were used to visualize and target the feedforward and feedback components of these intra-thalamic circuits and to understand how peripheral input from the retina impacts their development.

Retrograde tracing of thalamocortical (TC) afferents through TRN revealed that the modality-specific organization seen in the adult, is present at perinatal ages and seems impervious to the loss of peripheral input. To examine the formation and functional maturation of intrathalamic circuits between the visual sector of TRN and dLGN, we examined when projections from each nuclei arrive, and used an acute thalamic slice preparation along with optogenetic stimulation to assess the maturation of functional synaptic responses. Although thalamocortical projections passed through TRN at birth, feedforward axon collaterals determined by vGluT2 labeling, emerged during the second postnatal week, increasing in density through the third week. Optogenetic stimulation of TC axon collaterals in TRN showed infrequent, weak excitatory responses near the end of week 1. During weeks 2–4, responses became more prevalent, grew larger in amplitude and exhibited synaptic depression during repetitive stimulation. Feedback projections from visual TRN to dLGN began to innervate dLGN as early as postnatal day 2 with weak inhibitory responses emerging during week 1. During week 2–4, inhibitory responses continued to grow larger, showing synaptic depression during repetitive stimulation. During this time TRN inhibition started to suppress TC spiking, having its greatest impact by week 4–6. Using a mutant mouse that lacks retinofugal projections revealed that the absence of retinal input led to an acceleration of TRN innervation of dLGN but had little impact on the development of feedforward projections from dLGN to TRN. Together, these experiments reveal how and when intrathalamic connections emerge during early postnatal ages and provide foundational knowledge to understand the development of thalamocortical network dynamics as well as neurodevelopmental diseases that involve TRN circuitry.

**Supplementary Information:**

The online version contains supplementary material available at 10.1186/s13064-024-00183-5.

## Background

**Fig. 1:**
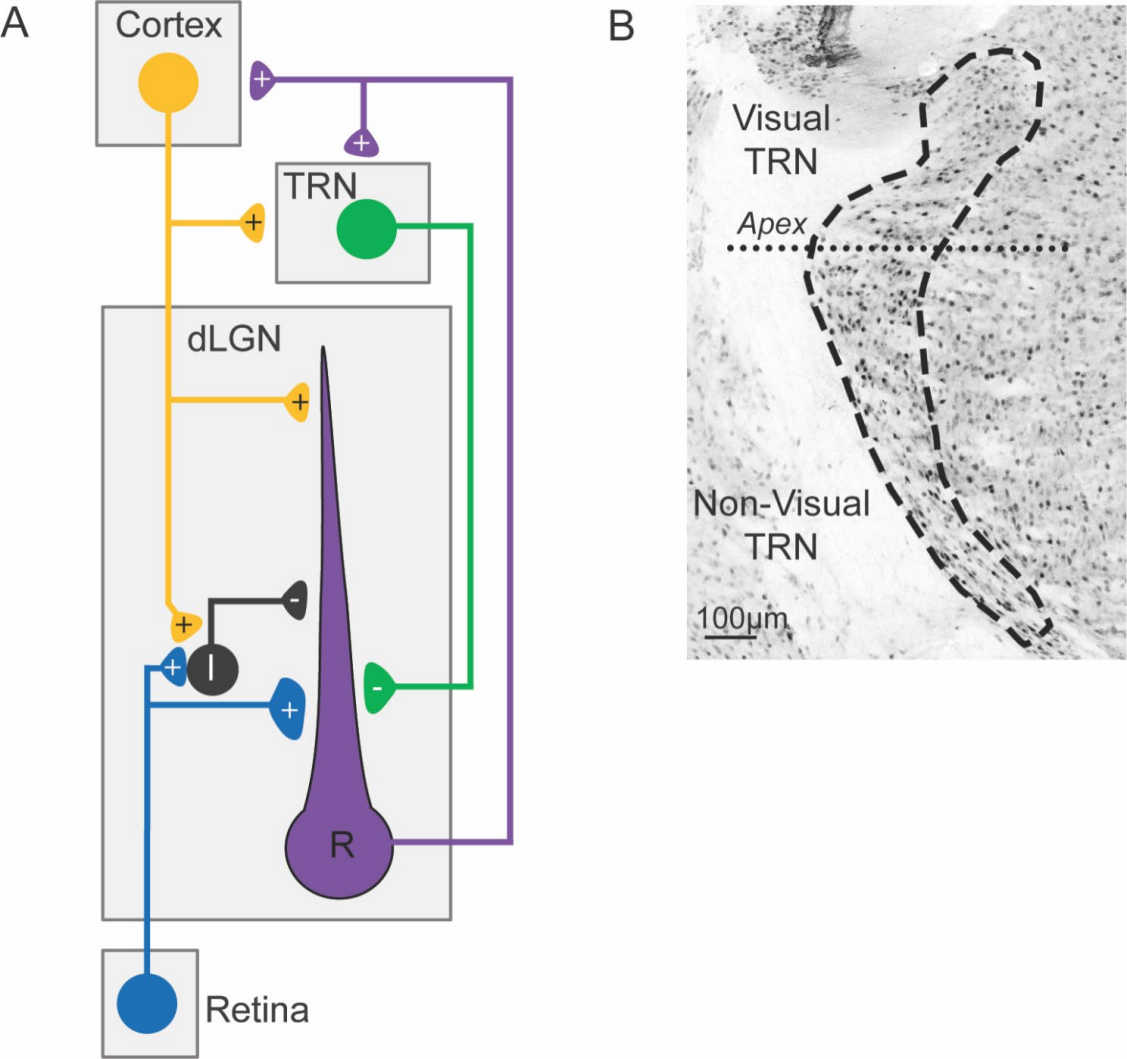
(**A**) Wiring diagram illustrating the pattern of connectivity within the visual thalamus. Retinal projections (blue) innervate the dorsal lateral geniculate nucleus (dLGN) and provide excitatory drive for intrinsic interneurons (black) and thalamocortical relay neurons (R, purple). Thalamocortical relay neurons convey excitatory signals to the visual cortex (yellow). Their axons pass through the visual sector of the thalamic reticular nucleus (TRN, green) and give off axon collaterals that excite TRN neurons (feedforward excitation). Neurons from visual TRN project back to dLGN relay neurons to suppress thalamocortical transmission (feedback inhibition). (**B**) NeuN staining in a coronal section from an adult mouse illustrates the cytoarchitectural borders (dashed lines) of the TRN. The apex (dotted line) is a landmark that delineates the division between visual and non-visual sectors of TRN [44, 74]

The thalamic reticular nucleus (TRN) is a shell-like structure that surrounds the dorsal and lateral aspects of the thalamus [1–5]. Comprised entirely of GABAergic neurons, the TRN serves as an important nexus for thalamocortical (TC) and corticothalamic (CT) communication [4, 6–9]. This nucleus receives excitatory input from thalamocortical and corticothalamic axon collaterals, which in turn serve to activate GABAergic inhibitory feedback to many thalamic nuclei. This pattern of connectivity is arranged in a sectorial manner that allows for both modality-specific and network-wide interactions (Fig. 1).

The TRN plays a key role in TC function by modulating sensory signaling during different behavioral states, participating in the generation and propagation of thalamocortical rhythms during sleep and wakefulness [3, 10, 11]. Additionally, a disruption in connectivity or in the intrinsic membrane properties of TRN neurons has been implicated in a number of neurodevelopmental diseases including epilepsy, autism spectrum disorder and attention hyperactivity disorder [9, 12–20]. Although the organization of TRN and its impact on state dependent behavior has been studied extensively, we still lack a fundamental understanding of how and when these intrathalamic connections develop and become operational. Studies in rodent have begun to characterize early postnatal TC network activity [21–23], however, the underlying circuitry linking the TRN to first-order thalamic nuclei remain unexplored.

The dLGN of the mouse has emerged as a model system to study thalamic circuit development [24]. Much of our present understanding is based on the study of the retinogeniculate pathway [24–26]. Studies have also delineated when and how input from nonretinal sources, such as those arising from layer VI of visual cortex, as well as those originating from cholinergic nuclei of the brainstem, innervate dLGN and form functional connections with thalamocortical relay neurons [27–30]. Taken together, these studies reveal that circuit assembly in dLGN is a highly orchestrated event, with retinogeniculate connections emerging first, followed by CT innervation during the second postnatal week, and then finally by input from cholinergic brainstem areas which proceeds slowly through the end of the first postnatal month. Interestingly, the arrival of retinal afferents plays a key role in the timing of nonretinal innervation. For example, the absence or elimination of retinal input accelerates the arrival of CT input by disrupting the expression of aggrecan, a repulsive chondroitin sulfate proteoglycan (CSPG) that normally inhibits cortical axons from entering dLGN prematurely [28]. However, the emergence of connections between dLGN and TRN have yet to explored in this context. More specifically, what is lacking is an understanding about how and when the connections linking dLGN and TRN occur, and whether the absence of retinal input affects the development of these feedforward and feedback loops. To accomplish this, we made use of specific Cre-driver, reporter, and transgenic lines that allow for the visualization and interrogation of intrathalamic feedforward and feedback circuits. To study the impact of retinal innervation, we took a loss of function approach and employed a novel form of genetic deafferentation by utilizing a Math5 null (Math 5^−/−^) mouse. This mutant lacks the transcription factor Math5, which is essential for the differentiation of retinal progenitors into retinal ganglion cells. As a result, Math5^−/−^ mice exhibit a wholesale loss (> 95%) of retinal ganglion cells, a failure to develop an optic nerve, and a brain that is devoid of retinofugal projections [31–34]. Finally, because our focus is on visual intrathalamic circuits, we also assessed whether the modality-specific sectors of TRN were present at birth and if such an arrangement was altered by visual deafferentation.

## Materials and methods

### Subjects

Experiments were conducted in mice P0-P46 of either sex. We used the GAD65 EGFP transgenic strain that expresses enhanced green fluorescent protein (EGFP) in TRN [27, 35, 36]. We also utilized two Cre-driver lines, somatostatin-Cre (SST-Cre; Jax stock no. 013044, RRID: IMSR_JAX:013044) and corticotropin releasing hormone-Cre (CRH-Cre, MMRRC no. 030850-UCD, RRID: MMRRC_030850-UCD), to target reporter expression within TRN (SST-Cre) or thalamocortical neurons (CRH-Cre). The Cre-driver lines were crossed with reporters to enable Cre-dependent expression of either tdTomato (Ai9; Jax stock no. 007909; RRID: IMSR_JAX:007909) or channelrhodopsin 2 – EYFP (ChR2; Ai32; Jax stock no. 012569; RRID: IMSR_JAX:012569). All lines were then crossed onto a Math 5^−/−^ background in order to study TRN and dLGN circuit assembly in the absence of retinal input [31, 33, 37]. All breeding and experimental procedures were approved by the University of Louisville Institutional Animal Care and Use Committee.

### Cholera toxin subunit B injections

To label projections passing through the TRN, P1 CRH-Cre or CRH-Cre x Math 5^−/−^ mice were deeply anesthetized using isoflurane vapors. Neonatal mice were placed in a small anesthesia induction chamber and initially anesthetized with 0.5–1.5% isolflurane in 100% oxygen. During the surgical procedure (< 5 min) anesthesia was maintained (0.5-1.0% isoflurane) by placing a small custom mask over the snout. Anesthetic depth was monitored by observing slowed abdominal instigation during breathing, loss of movement, and response to toe or limb pinch.

We stabilized the mouse head and made injections using a stereotaxic device that could clasp a pipette holder. We based our injection sites on an atlas of the developing mouse brain [38] using the rostral-caudal coordinates as well as the distance from midline. The skull was pierced with a sterile needle and then a glass pipette (10–20 μm tip diameter) filled with a 1% solution of Cholera toxin subunit B (CTB) conjugated to different Alexa Fluors (488, 546 or 647; Invitrogen) dissolved in distilled water was lowered into the targeted region. The pipette was attached to a picospritzer and 2uL of CTB was injected into visual cortex or somatosensory cortex. After a 24-hr survival period, animals were deeply anesthetized by isoflurane vapors and transcardially perfused with PBS followed by 4% paraformaldehyde in 0.1 M phosphate buffer (4% PFA). Brains were removed and post-fixed for 24 h in 4% PFA and then transferred to phosphate buffered saline [PBS: 0.01 M phosphate buffer (PB) with 0.9% NaCl]. To verify the injection site, the cortical surface of excised brains was imaged using a stereomicroscope (Olympus SZX2-ILLB) with fluorescence illumination (Prior Scientific Lumen 200).

### Immunohistochemistry

To visualize thalamocortical axon collateral terminals in TRN we used vesicular glutamate transporter 2 (vGluT2) labeling [39–41]. At least three coronal sections (35 μm) containing the visual sector of TRN from at least two different mice were collected from CRH-Cre and CRH-Cre x Math 5^−/−^ at ages that ranged from P5-P28. Sections were blocked (10% normal goat serum [NGS; Vector Labs, S-1000, RRID: AB_2336615] and 0.3% Triton X-100 in PBS) for 1 h and then incubated overnight in rabbit anti-vGluT2 (SysSys, AB135403 1:500, RRID: AB_887883; diluted 1:100 with 10% NGS in PBS). After a wash in PBS, a secondary antibody, goat anti-rabbit 488 (ThermoFisher, A11034, RRID: AB_2576217, 1:100, diluted in 1% NGS in PBS) was applied for 1 h. Sections were washed, then immunolabeled to visualize neurons, and incubated overnight in mouse NeuN (Milipore, MAB377, RRID: AB_2298772, 1:100; diluted in 1% NGS in PBS). After a wash, sections were incubated for 1 h with biotinylated goat anti-mouse (Vector Labs, BA9200, RRID: AB_2336171, 1:100, diluted in 1% NGS in PBS) and then labeled with streptavidin-AF647 (ThermoFisher, S21374, RRID: AB_2336066, 1:100 diluted in PBS). Sections were mounted with Prolong Gold (Invitrogen, P36931), coverslipped, imaged using confocal microscopy, and analyzed using methods described below.

For all in vitro recordings, biocytin (0.5%, Sigma) was included in the internal pipette solution for intracellular filling and 3D neuron reconstruction using confocal microscopy [33, 42, 43]. Following the completion of the recording session, slices were fixed overnight in 4% paraformaldehyde in 0.1 M phosphate buffered saline (PBS), then washed with PBS and incubated overnight with AlexaFluor 647-conjugated streptavidin (Invitrogen, S21374) in a PBS solution containing Triton X-100 (0.1%).

### Image acquisition and analysis

To visualize TRN terminals in dLGN, and TC axons in TRN, fixed brains from GAD-65 EGFP and CRH-Cre X Ai9 mice were cut in the coronal plane (70 μm) and mounted on slides using ProLong mounting medium containing DAPI (Life Technologies P36931). High contrast images and DAPI staining were used to assist in delineating the borders of dLGN. Sections containing TRN or dLGN were imaged using a multiphoton laser scanning confocal microscope (Olympus, model no. FV1200BX61) equipped with a 20 × (0.75 NA) objective. Fluorophores were excited using Ar (488 nm) and HeNe (635 nm) lasers, and Z-stacked images (1.26 μm optical sections) were acquired using Fluoview software at a scanning resolution of 1600 × 1600 pixels.

To quantify the spatial extent of innervation or the density of VGlut2 staining at different postnatal ages, we analyzed at least three coronal sections through the middle of TRN or dLGN [29, 30, 44]. Z-stacks of each image were generated and imported into Photoshop (Adobe). Images were binarized using a threshold that clearly distinguished signal and background fluorescence. Binarized images were imported into ImageJ (NIH, RRID: SCR_003070) in order to count the number of fluorescent pixels as well as the total number of pixels within a nucleus. These values were used to calculate the percent area that contained fluorescent pixels. At each postnatal age, values were obtained from 3 to 28 hemispheres taken from > 2 mice. For summary statistics, each hemisphere served as a unit of observation and the median value with a 95% confidence interval were plotted.

### In vitro slice preparation and whole cell recording

Acutely prepared, thalamic brain slices were made from mice that were deeply anesthetized and rapidly decapitated. The brain was excised and placed into 4 °C oxygenated cutting solution (in mM): Sucrose 234, glucose 11, NaHCO3 26, MgSO4 10, KCl 2.5, CaCl2 0.5, NaH2PO4 1.25. Brain slices containing thalamus were cut in the coronal plane (270 μm thick) on a vibratome (Leica), placed in a chamber and bathed for 30 min in warm (32 °C) oxygenated artificial cerebrospinal fluid (aCSF) (in mM: 126 NaCl, 26 NaHCO3, 10 glucose, 2.5 KCl, 2 MgCl2, 2 CaCl2, 1.25 NaH2PO4). Recordings were conducted in a chamber perfused continuously with 32 °C aCSF at a rate of 2–3 mL/min. Thalamic nuclei were visualized on an upright microscope (Olympus BX51WI) with DIC optics and fluorescent filters (GFP: Chroma 49,002; tdTomato: Chroma 49,005) using a 10x or 60x water immersion objective. A vertical puller (Narashige PC-10) was used to pull patch electrodes from borosilicate glass. For voltage clamp recordings, the electrode solution contained (in mM) 117 Cs gluconate, 11.0 CsCl, 1.0 MgCl2, 1.0 CaCl2, 0.1 EGTA, 10.0 HEPES, 2 Na2-ATP, 0.4 Na2-GTP. For current clamp recordings, the electrode internal solution contained (in mM) 117 K-gluconate, 13 KCl, 1.0 MgCl2 1, 0.07 CaCl2, 0.1 EGTA, 10 HEPES. The final electrode tip resistance was 4–7 M Ohms. Whole cell recordings were made in current or voltage clamp mode using an amplifier (Multiclamp 700B, Molecular Devices), filtered at 3–10 kHz, and digitized (Digidata 1440 A) at 20 kHz. Pipette capacitance, series resistance, input resistance, and whole-cell capacitance were monitored throughout the recording session. Inhibitory postsynaptic currents were measured by holding the neuron at 0mV using a cesium-containing electrode while excitatory currents were measured at -70mV using a potassium-containing electrode.

### Optogenetic stimulation and analysis

We took an optogenetic approach to assess functional connections between TRN and dLGN. By crossing CRH-Cre [45] or SST-Cre [36] with an Ai32 mouse [46–48], we were able to selectively stimulate feedforward or feedback projections in thalamic slices in WT and Math 5^−/−^ backgrounds. Several labs, including our own [36], make use of cell type specific Cre lines to visualize or interrogate TRN circuitry [12, 49–53]. These are usually done in conjunction with Cre-dependent reporter lines or Cre-dependent viral injections. While the latter is ideal for achieving cell type specificity, it falls short in providing widespread and sufficient expression in a target structure, a task more readily achieved by using Cre-driver lines. The TRN is a deep structure and especially difficult to target in neonatal brains. Thus, we chose to take a Cre-driver approach rather than a Cre-viral vector one in order to maximize the expression of ChR2 in an acute thalamic slice preparation [30, 36, 45, 54]. Thus, it opens up the possibility that off-target projections could contribute to our results. In the case of SST-Cre, according to transcriptomic expression databases [55, 56], the somatostatin gene used to drive Cre expression in TRN, is absent in mouse dLGN. While present in the cortex and superior colliculus, two structures that project to dLGN [24], SST is restricted to interneurons [55], a neuronal cell type that does not project to dLGN. Additionally, it is important to note that the CRH-Cre line we utilized here is the transgenic KN282 line developed by GENSAT [57]. The expression pattern of this Cre driver is distinct from the “knock-in” CRH-IRES-Cre line which does not display Cre expression in the thalamus [55]. When crossed onto a Cre-tdTomato reporter line (Ai9), expression was restricted to thalamocortical relay neurons residing in 1st and higher order thalamic nuclei [44, 45, 58]. Thus, while off-target interactions cannot be entirely ruled out, they are not likely to contribute to our reported results.

While the TRN contains PV and SST expressing neurons, we chose to target the SST population. The SST-Cre line has been previously described and used extensively for thalamic circuit dissection (50,51,52,53). Moreover, the majority of neurons in the visual sector of TRN are SST positive (36,52,54,55). Indeed, in a previous study, we obtained estimates of the number of SST expressing neurons in the visual sector of TRN by crossing SST-Cre with an Ai3 (EYFP) reporter line, and then using a NeuN antibody to determine the percentage of doubled-labeled neurons (36). We found that about 82% vTRN neurons were double labeled.

Light-gated postsynaptic responses were evoked using a light emitting diode (LED, Prizmatix) that delivered blue light through a 60x objective. Blue light pulses were 0.3 mm in diameter with a light power of 525mW/mm2 and with a pulse width of 1ms. Trains of 10 blue light pulses were presented at different temporal frequencies (0.25, 0.5, 1, 5, 10 and 50 Hz). The incidence of optically evoked postsynaptic responses was assessed by determining if the amplitude was > 2 x RMS (root mean squared) of baseline [44]. The peak amplitude was measured relative to baseline levels obtained for 1s just prior to photostimulation. To examine the changes in the synaptic response to repetitive stimulation, paired pulse ratios were calculated by comparing the amplitude of the initial response to that evoked by the nth pulse (EPSCn/EPSC1) as well as the 10th pulse (EPSC10/ EPSC). To examine how TRN stimulation affects spiking activity, dLGN neurons were held at -70 mV and injected with a 1.5-s square wave depolarizing current pulse of sufficient strength (45pA-250pA pA) to evoke a steady train of spike firing *≥* 5 Hz [36]. Changes in spike firing were then calculated by comparing equivalent periods (0.5 s) of activity in the presence or absence of blue light stimulation (2, 5, 10, 20, and 50 Hz).

Typically, the measurements described above were based on the average of four stimulus presentations. All traces reflect the averaged responses of individual trials. All statistical tests were performed using Prism 10.1.1 (Graphpad Software, La Jolla, CA) and reported in the results section.

## Results

### Development of the sectorial arrangement of TRN

**Fig. 2:**
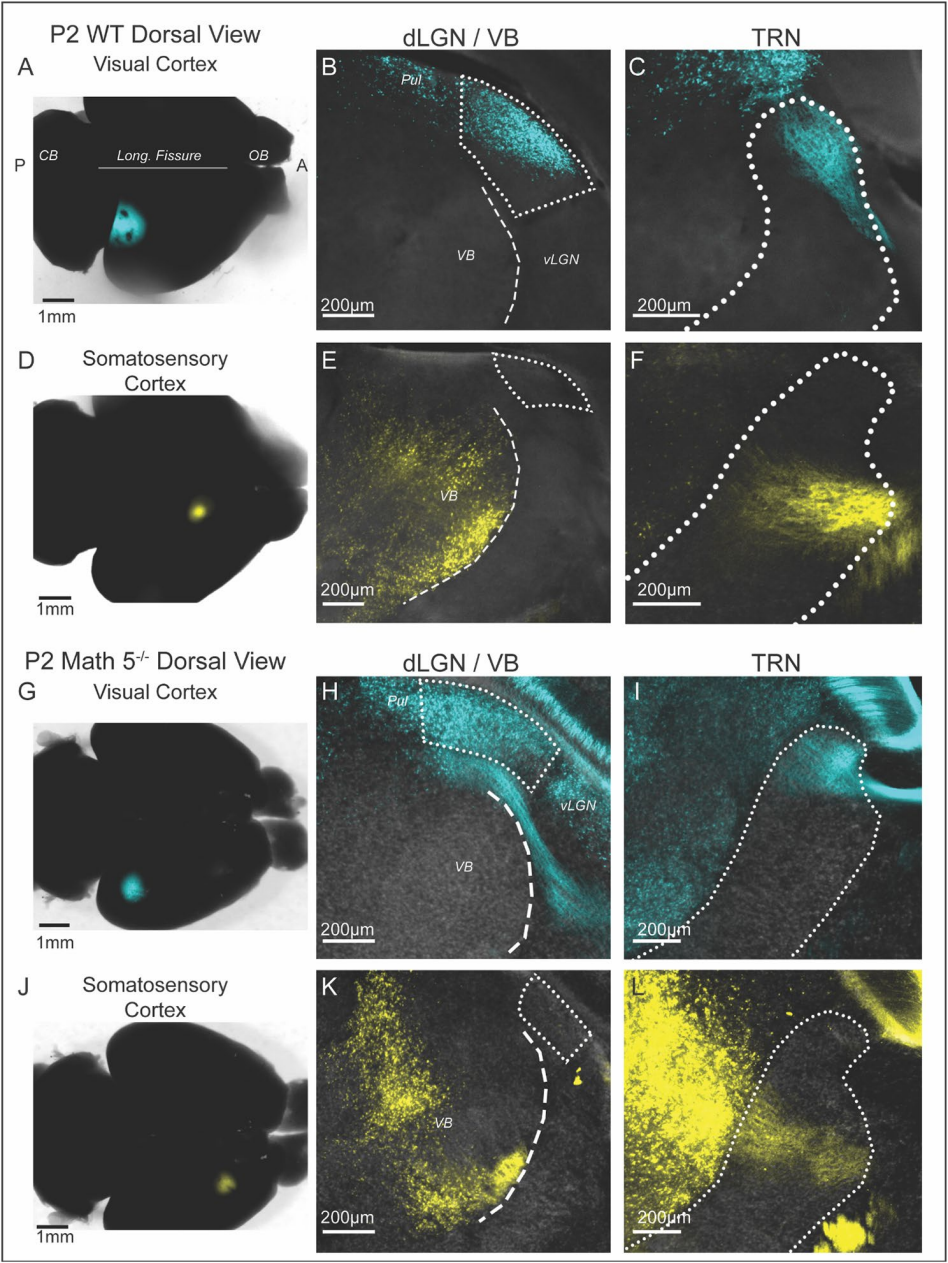
Identification of TRN sensory sectors in P2 WT and Math 5^−/−^ mice. (**A**) An image of the cortical surface of a P2 brain illustrating the location of the CTB injection into the visual cortex. Coronal sections from this case are shown in B and C. Dotted line depicts the longitudinal fissure. Abbreviations: CB: cerebellum, OB: olfactory blub, Pul: pulvinar, VB: ventrobasal complex, A: anterior, P: posterior. (**B**) Retrograde labeling of thalamocortical (TC) neurons in dLGN (outlined by dotted lines). Some TC neurons in Pul were labeled, but VB and medial geniculate nucleus (MGN, not shown) were devoid of CTB. (**C**) Coronal section through TRN (outlined by dotted lines) shows CTB-labeled TC projections restricted to the head of TRN. There was an absence of CTB labeling below the apex. (**D**) The cortical surface of a P2 brain illustrates the location of a CTB injection into somatosensory cortex (yellow). Coronal sections from this case are shown in **E** and **F**. (**E**) Retrograde labeling of TC neurons was seen throughout much of VB, but no CTB was observed in dLGN. (**F**) CTB-labeled TC projections from VB were detected at and below the apex of TRN. (**G**) The cortical surface of a P2 Math 5^−/−^ brain showing a CTB (blue) injection into the visual cortex. Coronal sections from this case are shown in **H** and **ID**. (**H**) Retrograde labeling of TC neurons was prevalent in dLGN and adjacent pulvinar nucleus but absent in VB (ventral to dLGN) or vMGN (not shown). (**I**) TC axons passing through TRN were restricted to the head of TRN, dorsal to the apex. (**J**) The cortical surface of a P2 Math 5^−/−^ brain illustrating a CTB injection (yellow) into the somatosensory cortex. Coronal sections from this case are shown in **K** and **L**. **K.** TC neurons in VB were retrogradely labeled by CTB but none were found in dLGN. **L** In TRN, TC labeled axons were confined to regions ventral to the apex

The TRN is comprised of nonoverlapping, modality-specific sectors that are defined by the source of their ascending thalamocortical (TC) and descending corticothalamic (CT) collateral inputs [3, 44, 59–61]. While TC and CT projections pass through TRN at perinatal ages [62–67], it remains unclear whether they innervate the TRN in a segregated, modality-specific manner. To address this, we made injections of (CTB conjugated to either 488 or 647 Alexa dyes) into visual (Fig. 2, blue) or somatosensory cortex (Fig. 2, yellow) in WT mice at postnatal day 1 (*n* = 6). As early as P2, CTB injections into visual cortex (Fig. 2A) resulted in retrograde labeling of TC neurons in dLGN as well as the pulvinar, but not in ventrobasal complex (VB) (Fig. 2B). In all six cases, the labeled axons within TRN were restricted to the head (i.e., dorsal-caudal region) of the nucleus (Fig. 2C, see Fig. 1B). Moreover, we failed to detect any axonal labeling ventral to the apex of TRN, a boundary that delineates visual and nonvisual sectors of the TRN in the adult mouse [44]. A similar modality specific patterning was evident following CTB injections into somatosensory cortex (Fig. 2D). These injections labeled TC neurons that were restricted to somatosensory thalamic nuclei such as the ventroposteromedial (VPM) and ventroposterolateral (VPL) nuclei (i.e., ventrobasal complex (VB), Fig. 2E). In TRN, the axons from somatosensory nuclei were restricted to the ventro-medial region at or below the apex (Fig. 2F) adjacent to VB. Thus, based on the coarse labeling pattern observed in TRN, it appears that visual and somatosensory axons of passage are arranged in a largely nonoverlapping manner.

It should be noted that while CTB injections led to somatic labeling in thalamus, indicative of retrograde transport, the axonal labeling observed in TRN could also include corticothalamic fibers that were labeled in an anterograde manner [68]. Indeed, during early development these fiber systems course together in opposing directions through pallial- subpallial boundary, the internal capsule and TRN, working together to assist each other in their respective guidance and targeting [63, 69].

While TRN sectors are established at perinatal ages, it is unclear whether this arrangement is preserved in the absence of peripheral sensory input. To test for this, we made similar CTB injections in Math 5^−/−^ mice, a mutant mouse that lacks > 95% of retinal ganglion cells and that is completely devoid of retinal input to the brain [31, 33, 37]. Similar to WT, injections into visual cortex of Math 5^−/−^ mice (Fig. 2G and H) led to retrograde labeling in the head of TRN above the apex (Fig. 2I, blue, *n* = 3), while injections into somatosensory cortex (Fig. 2J and K) labeled axons in the ventromedial region of TRN at or below the apex (Fig. 2L, yellow, *n* = 2).

Taken together, these results reveal that the sensory specific organization of TRN is established by perinatal ages and that such organization is retained in the absence of sensory stimulation. These results also allowed for us to examine the formation of visual feedforward and feedback connections by targeting the dorsal caudal aspect (head) of TRN (see Fig. 1B).

### dLGN innervation of TRN

**Fig. 3:**
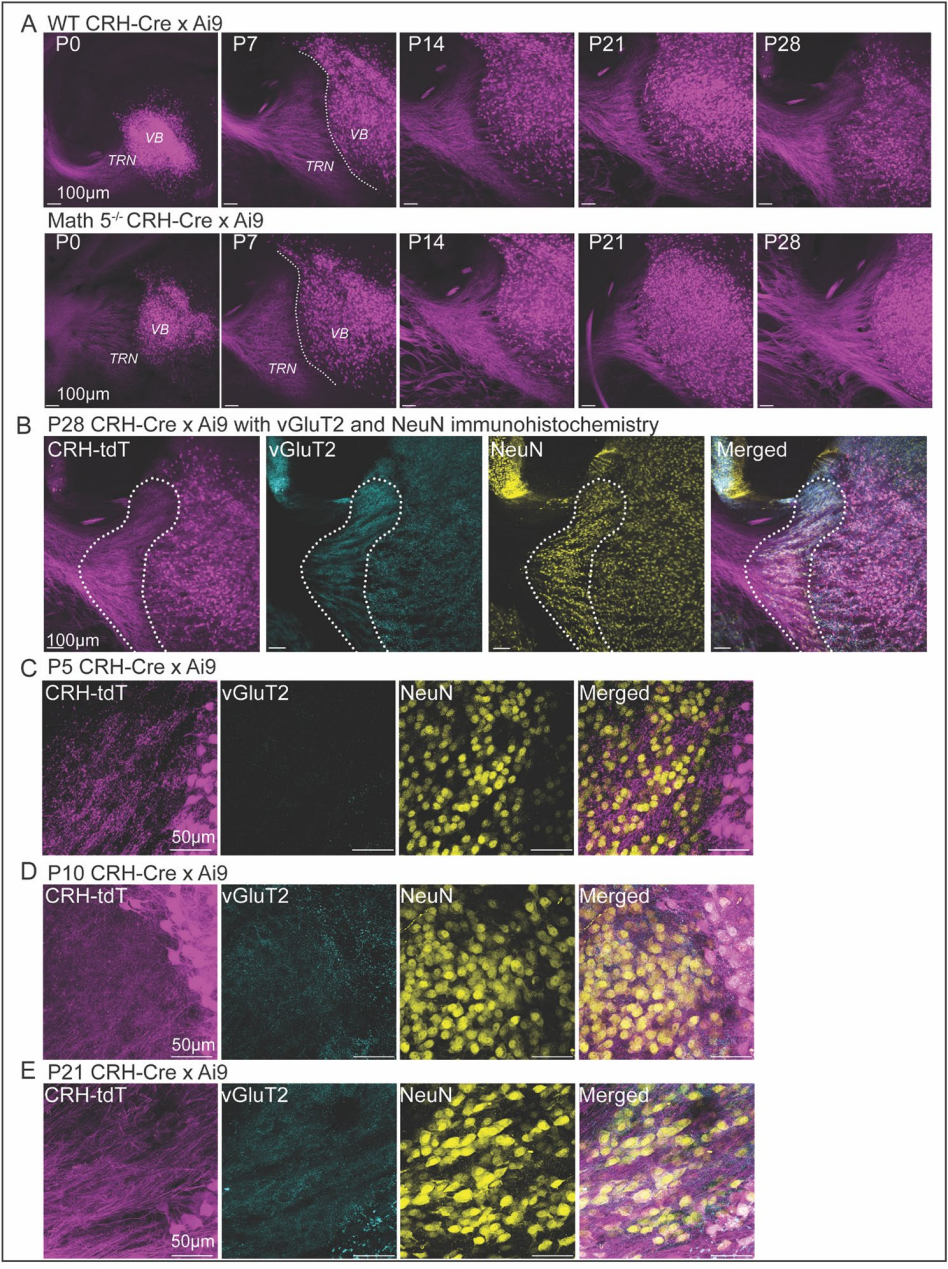
Visualization of TC axons and their collateral terminals in TRN. (**A**) Thalamocortical (TC) projections coursing through TRN in CRH-Cre x Ai9 (tdtomato) WT and CRH-Cre x Ai9 mice crossed onto a Math 5^−/−^ background. Coronal sections from CRH-Cre x Ai9 WT (top panels) and CRH-Cre x Ai9 Math 5^−/−^ mice (bottom panels) at postnatal day (P) 0, P7, P14, P21 and P28. At all ages and for both groups, robust td-tomato was evident in TC neurons located in ventrobasal complex (VB) as well as TC axons coursing through the TRN in route to neocortex. Dotted line delineates the border between TRN and VB. Scale bar = 100 μm. (**B**) Coronal sections through TRN (outlined by dotted lines) from a P28 CRH-Cre x Ai9 mouse with vGluT2 immunohistochemistry and NeuN labeling. Panels from left to right: Cre-dependent tdTomato (tdT, purple) labeled TC neurons and their axons coursing through TRN. vGluT2 immunohistochemistry (blue) labeled TC terminals in TRN. NeuN (yellow) is a marker of somatic nuclei and used to delineate the borders of TRN. Merged image showing the labeling pattern of TC axons, vGluT2 containing terminals, and NeuN somata. C-E. High power images showing the tdTomato labeled TC axons, vGluT2 labeled terminals and NeuN labeled somata for CRH-Cre x Ai9 WT at P5 (**C**), P10 (**D**) and P21 (**E**). Scale bar C-E = 50 μm

To visualize excitatory feedforward projections from dLGN to TRN, we crossed corticotropin releasing hormone-Cre (CRH-Cre) mice with an Ai9 reporter line [44, 58, 70] in order to express tdTomato in TC neurons of 1st order thalamic nuclei such as the dLGN and VB complex [44, 45, 58]. Figure 3 provides examples of coronal sections of labeled TC axons at different postnatal ages in both WT (Fig. 3A) and Math 5^−/−^ (Fig. 3B) mice. For both groups, as early as P0, TC axons coursed through TRN on a trajectory from the medial aspect of the nucleus to the lateral edge where they aggregate to form the internal capsule. At later ages, axons within TRN were arranged into fascicles forming a reticular configuration. This arrangement emerged as early as P7 and was readily apparent within the head of vTRN by P14.

It is important to note that the projections from dLGN to TRN involve fine caliber axon collaterals while thalamocortical axons are much larger and bundled into fascicles [3]. Indeed, these processes are difficult to resolve at the light microscopic level. Thus, to visualize terminal boutons of thalamocortical axon collaterals in TRN we used an antibody against a vesicular glutamate transporter 2 (vGluT2) [39–41, 71, 72]. Figure 3C-E illustrates the punctate vGluT2 labeling of TC axon collaterals (blue) in relation to TC tdTomato axonal labeling (purple) and TC somatic NeuN labeling (yellow) in the same coronal section through the TRN of an adult mouse.

**Fig. 4:**
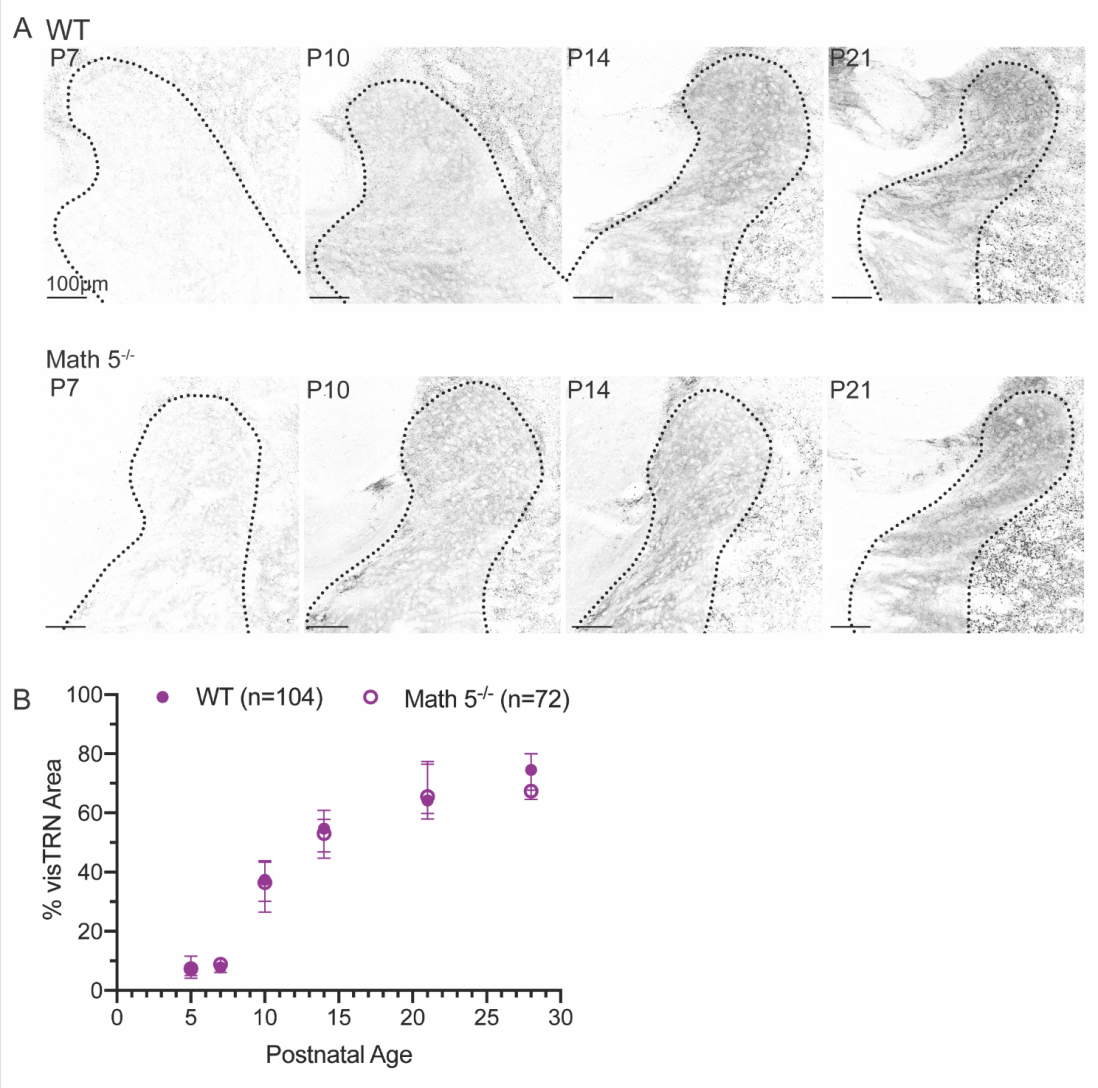
Visualization of TC axon terminals in TRN using vesicular glutamate transporter 2 (vGluT2) immunohistochemistry. (**A**) Coronal sections depict a progressive increase in vGluT2-positive TC terminals within TRN for WT (top) and Math 5^−/−^ (bottom) mice at P7, P10, P14 and P21. In both WT and Math 5^−/−^, vGluT2 labeling was nearly absent at P7 (left) with labeled puncta emerging by P10 and increasing in density with age. (**B**) Summary plot depicting the spatial extent of TC axon collateral terminals in visual TRN (regions above the apex) as a function of postnatal age. Each data point represents the median percent of visual TRN area covered by vGluT2 labeling (± 95% confidence interval) in both WT (solid symbols, *n* = 104 sections) and Math 5^−/−^ (open symbols, *n* = 72 sections) mice. Values were derived from *≥* 8 hemispheres from *≥* 2 animals using 2–3 successive sections of visual TRN. The area covered by vGluT2 labeling increased with age for both WT and Math 5^−/−^ mice

To determine when TC axon collateral terminals appear in vTRN and whether they are affected by the removal of retinal input, we examined vGluT2 labeling in coronal sections from age-matched WT (Fig. 4A, top) and Math 5^−/−^ (Fig. 4A, bottom) mice. To analyze the rate of TRN innervation by TC axon collaterals, we quantified the percentage of vTRN area covered by vGluT2 puncta across different postnatal ages and plotted the median values (Fig. 4C; [44]). Measurements were confined to the head of TRN above the apex (Fig. 1B.) In WT, the area covered by vGluT2 puncta increased with age (filled circles, *n* = 104 sections, Kruskal-Wallis: *p* < 0.0001). During the first postnatal week (P5-P7) vGluT2 labeling was sparse but showed a progressive increase, eventually covering nearly half of vTRN by P10 (P5 and P7 vs. P10, Dunn’s multiple comparison test, *p* < 0.01) and then all of it by P21 (all comparisons between P10, P14 and P21, *p* < 0.01). At P21, thalamic innervation of visual TRN plateaued and did not differ from P28 (*p* = 0.3063). Thus, innervation of vTRN by TC axon collaterals occurred largely during the second postnatal week (P7-P14) and encompassed the entire visual sector by the end of the third postnatal week (P21). Similar to WT, there was an age-related increase in vGluT2 puncta in the vTRN of Math 5^−/−^ mice (Fig. 4C, open circles, *n* = 72 sections, Kruskal-Wallis: *p* < 0.0001). Moreover, the age-related increase in vGluT2 labeling had a similar trajectory in WT and Math 5^−/−^ mice (two-way ANOVA, F_genotype (1,164)_ = 0.5503, *p* = 0.4593). Together, these data demonstrate that while TC axons course through TRN at birth, the appearance of axon collaterals increases during the second postnatal week and reaches adult-like levels by the end of the third postnatal week (P21). Furthermore, the absence of retinal inputs does not alter the time course of TC innervation of visual TRN.

### TRN axon innervation of dLGN

**Fig. 5:**
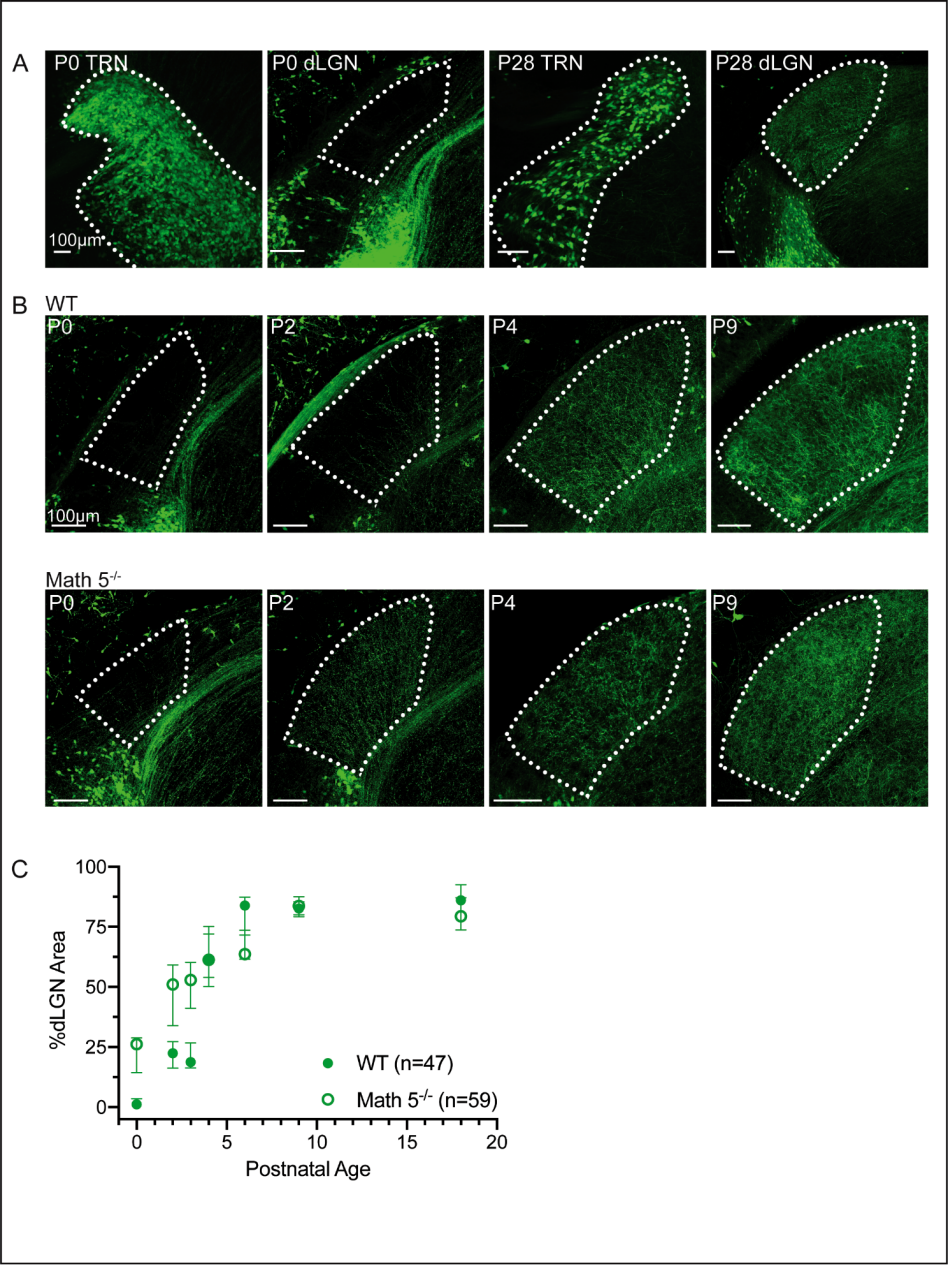
Development of TRN projections to dLGN in the presence and absence of retinal input. GAD65-EGFP mice were used to visualize TRN projections in WT. Math 5^−/−^ mutants crossed with GAD65-EGFP mice were used to examine projections in the absence of retinal innervation. (**A**) Examples of confocal images of TRN and dLGN at P0 (left) and P28 (right) from WT GAD65 EGFP mice depict robust labeling of TRN neurons. Note the lack of somatic EGFP expression in dLGN (see also  [27, 36]). (**B**) Coronal sections from early postnatal GAD65 mice containing the middle region of dLGN. Examples show the developmental time course of TRN innervation of dLGN both in the presence (WT, top) and absence (Math 5^−/−^, bottom) of retinal input. (**C**) Summary plot showing the percentage of dLGN area covered in fluorescent fibers as a function of postnatal age for both WT (solid symbols) and Math 5^−/−^ (open symbols). Each data point (green) is the median value of 47 WT and 59 Math 5-/- sections obtained from *≥* 3 hemispheres of *≥* 2 mice with error bars representing 95% confidence intervals

To visualize inhibitory feedback projections from TRN to dLGN we used GAD65-EGFP mice [27, 35, 36], which labels TRN neurons with EGFP as early as P0 (Fig. 5A). We also crossed Math 5^−/−^ mutants with GAD65-EGFP mice to assess whether the absence of retinal input disrupted the timing of feedback innervation.

Figure 5 provides coronal sections of dLGN from early postnatal GAD65 and GAD65 x Math 5^−/−^ mice. In WT (Fig. 5B, top row), TRN fibers were absent from dLGN at birth, but began to innervate the nucleus at P2 from the medial-ventral border. By the end of the first postnatal week TRN input encompassed all of dLGN in a dense plexus of terminals. We analyzed the rate of TRN innervation by quantifying the spatial extent of terminals expressed as a percentage of dLGN area [29, 30] and plotted the median values (Fig. 5C) for WT and Math 5^−/−^ groups. In WT (filled circles, *n* = 47 sections), TRN terminals arrived in dLGN at P2 and showed a progressive increase with age (Kruskal-Wallis, *p* < 0.0001). Most of the projections arrived between P2-4 and showed nearly a 3-fold increase over this period (median values, P2 = 22.40 vs. P4 = 61.34). By P6, TRN innervation was complete, forming a dense but diffuse network that spanned the entire nucleus (median values, P6 = 83.87, P9 = 82.67, P18 = 86.08). In Math 5^−/−^ (*n* = 59 sections). The absence of retinal input appeared to accelerate TRN innervation of dLGN (Fig. 5B bottom row). At P0, TRN axons could be seen innervating the ventral medial border, and by P2 they nearly encompassed all of dLGN with some axons extending close to the dorsal lateral border just beneath the optic tract. TRN terminals showed a two-fold increase in innervation between P0 and P2, (open circles *n* = 59) which rose steadily between P4-6 (median values: P0 = 26.18, P2 = 51.02, P4 = 61.28). This pattern of innervation was significantly different from WT mice showing greater innervation between P0-3 (multiple K-S tests, for P0, P2 and P3, D = 1.000, adjusted p-value = < 0.05). Together, these data demonstrate that TRN projections arrive in dLGN during the first postnatal week and that loss of retinal input accelerates innervation but does not seem to alter the overall density or pattern of innervation.

### Development of feedforward synaptic connections between dLGN and TRN

We adopted an optogenetic approach to assess when functional feedforward connections between dLGN and TRN neurons emerge and to test whether this time course was influenced by the loss of retinal input. To photoactivate TC axon collateral terminals and record postsynaptic responses in TRN neurons, we used CRH-Cre x Ai32 (ChR2-EYFP) mice and crossed these onto a Math 5^−/−^ background. To determine if ChR2 expression in TC neurons was sufficient to drive postsynaptic activity in TRN at early postnatal ages, we recorded light evoked activity directly from TC neurons. At P4, ChR2-EYFP was evident in TC neurons, and blue light stimulation with brief pulses (1ms) led to light evoked depolarizations and spike firing (Figure S1A-B).

To examine feedforward excitatory postsynaptic activity in TRN, we presented repetitive trains of blue light at different temporal frequencies (0.25 Hz, 0.5 Hz, 1 Hz, 5 Hz, 10 Hz, 50 Hz) and conducted voltage clamp recordings using a potassium-based internal solution while holding vTRN neurons at -70mV. We recorded from 186 WT and 114 Math 5^−/−^ neurons between P5-28 that were verified to be in vTRN based on their biocytin filled reconstructions.

**Fig. 6:**
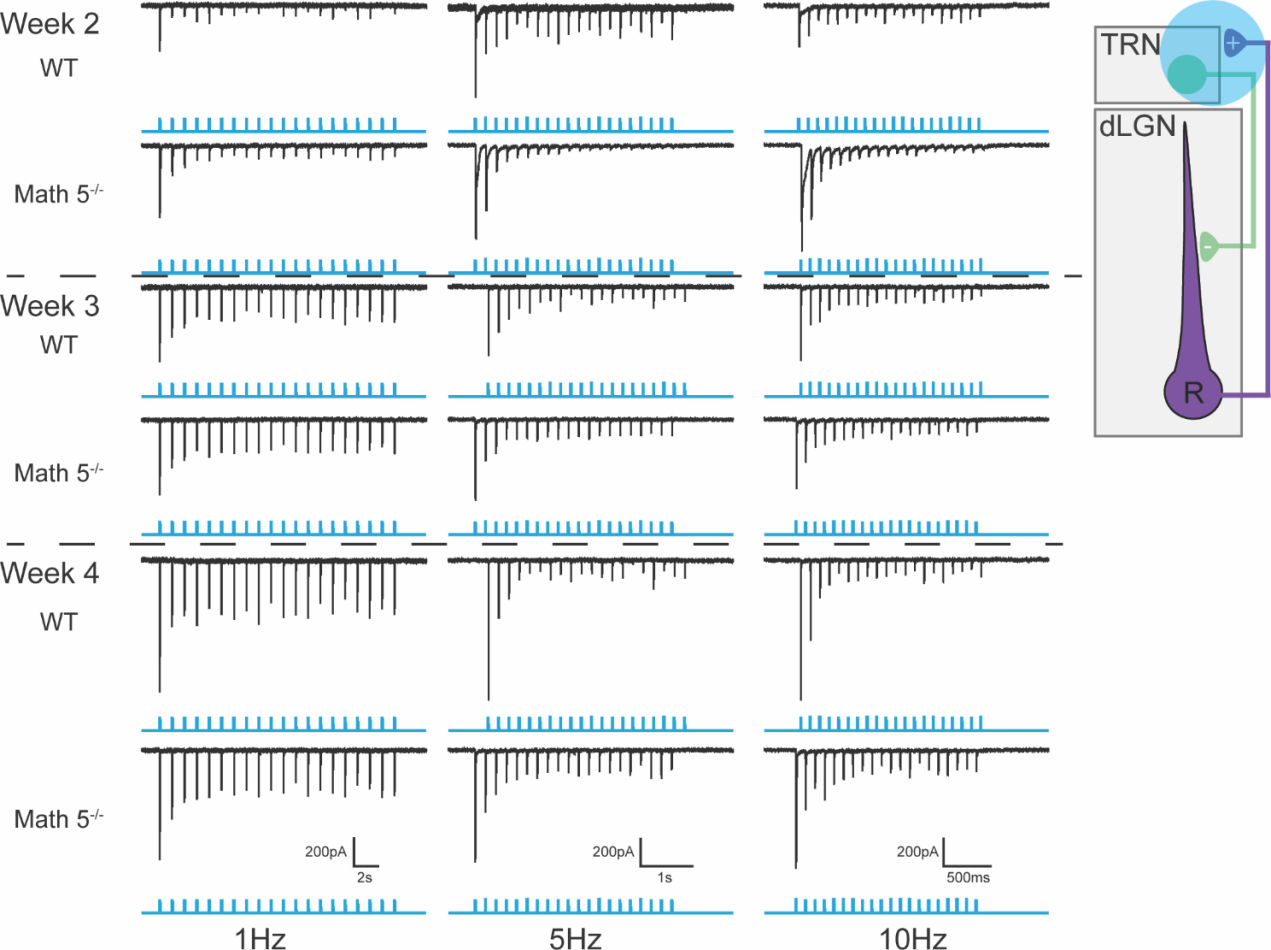
Light evoked synaptic responses of TRN neurons from WT and Math 5^−/−^ CRH-Cre x Ai32 (ChR2-EYFP) mice. Examples of excitatory synaptic responses recorded in voltage clamp mode to trains of blue light (20, 1ms pulses delivered at 1, 5 and 10 Hz) are depicted by postnatal week for WT and Math 5^−/−^ mice. Trains of blue light are shown in blue beneath each corresponding response. The inset depicts feedforward (purple) and feedback green circuits between TRN and dLGN and the blue circle indicates the location of blue light stimulation. All neurons held at -70mV

**Fig. 7:**
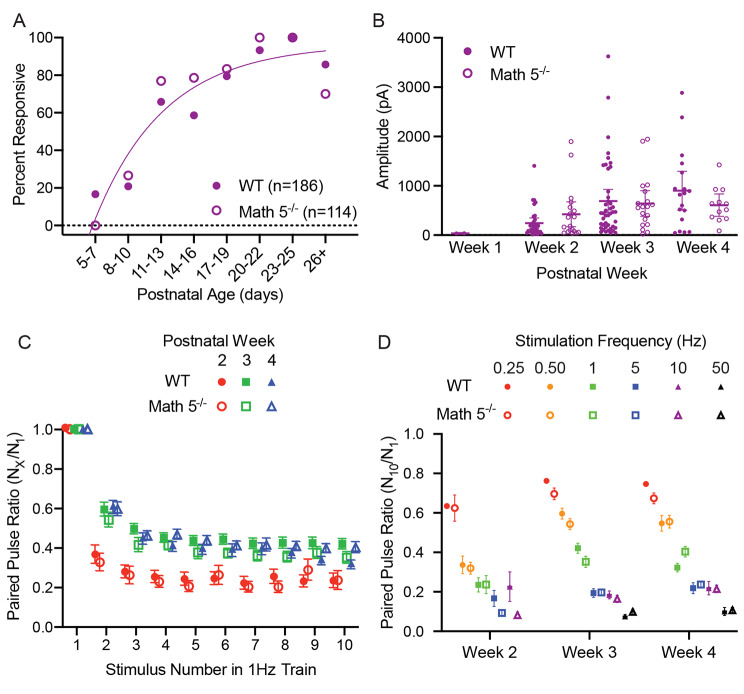
Summary of the light evoked synaptic responses of TRN neurons from WT and Math 5^−/−^ CRH-Cre x Ai32 (ChR2-EYFP) mice. (**A**) Plot of the percent of responsive cells by age for WT (solid symbols, *n* = 186) and Math 5^−/−^ (open symbols, *n* = 114) mice. Each point represents data from 4–39 neurons (median = 15). For both groups, incidence increased with age, nearing 100% by P20. (**B**) The amplitude of the initial (maximal) EPSC is plotted by postnatal week in WT (solid symbols, *n* = 96) and Math 5^−/−^ (open symbols, *n* = 50). Bars represent the mean amplitude (± SEM) at each postnatal week. EPSC amplitude increased with age. (**C**) A plot of the paired pulse ratios as a function of stimulus number for a 1 Hz stimulus train. Ratios of EPSC amplitudes were calculated by dividing the n^th^ response by the initial response. Each point represents the mean (± SEM) of at least 14–43 neurons. PPRs < 1 reflect synaptic depression. Symbols depict different weeks with solid ones representing WT and open ones Math 5^−/−^ groups. Both groups showed comparable degrees of depression with the largest attenuation noted during week 2. (**D**) The paired pulse ratios of EPSC amplitudes during a 0.25, 0.50, 1, 5, 10 and 50 Hz Hz stimulus train was calculated by dividing the 10th response by the initial response. Each point represents the mean (± SEM) of at least 6–43 (median = 14) neurons. Temporal frequency of stimulation (0.25–50 Hz) is organized by color. Both age and stimulus frequency led to lower PPRs, which indicates a greater degree of synaptic depression

Examples of the light evoked responses to blue light pulse trains (1ms, 20 pulses) trains presented at 0.5 Hz, 5 Hz, and 10 Hz are shown in Fig. 6. During the first postnatal week (P5-7), photoactivation of TC axon collaterals in visual TRN evoked little postsynaptic activity. EPSC activity was rare, of low amplitude and could only follow low rates of stimulation (e.g., 0.5 Hz). Between the second and third postnatal weeks in both WT and Math 5^−/−^ mice, the incidence of light evoked EPSC activity was more prevalent, greater in amplitude and capable of following higher rates of stimulation (e.g., 10 and 50 Hz by week 3). A similar profile was observed during the fourth postnatal week with robust excitatory responses recorded throughout the stimulus train. Of notable significance was the emergence of synaptic depression when higher rates of stimulation were used. For example, by week 3, at 5 Hz and 50 Hz the amplitude of EPSCs began to attenuate after the initial pulse but then stabilized to values that were about half the amplitude of the first response. This form of synaptic depression can be quantified by generating paired pulse ratios where the amplitude of the N^th^ response within a stimulus train can be is divided by the initial response (EPSCn/EPSC1; see Fig. 7C-D).

A summary of these age-related changes for WT and Math 5^−/−^ mice are shown in Fig. 7, where we plot the incidence of responsive neurons (Fig. 7A), the amplitude of the initial (and maximal) response (Fig. 7B), and the paired pulse ratios (Fig. 7C EPSCn/EPSC1; Fig. 7D EPSC10/ESPC1). For both WT and Math 5^−/−^, during the first postnatal week, only a few neurons showed weak light evoked responses (P5-7, WT: 3/18, 17%; Math 5^−/−^: 0/8, 0%). However, between P11-13, the incidence of light evoked EPSC activity showed about a three-fold increase (P11-13, WT: 25/38, 66%; Math 5^−/−^: 20/26, 77%). The incidence of light evoked activity continued to rise so that by P21 nearly all neurons tested were responsive (P19-21, WT: 28/31, 90%; Math 5^−/−^: 16/18, 89%). This progression was unaffected by the loss of retinal input with Math 5^−/−^ mice showing a similar trajectory as WT (Fisher’s exact test, *p* > 0.05). Consistent with the increase in incidence, both groups showed an age-related increase in peak amplitude. Overall, amplitude increased with age (Fig. 7B, two-way ANOVA, F_age(2,138)_ = 5.743, *p* = 0.0040) and was unaffected by the loss of retinal input (F_genotype(1,138)_ = 0.2572, *p* = 0.6128). In WT and Math 5^−/−^, peak amplitude increased during the second postnatal week but was stable between weeks 3 and 4 (Tukey’s multiple comparison test, week 2 vs. week 3: *p* = 0.0174; week 2 vs. week 4: *p* = 0.0096; week 3 vs. week 4: *p* = 0.7982).

To estimate the degree of synaptic depression, we computed paired pulse ratios (Fig. 7C EPSCn/EPSC1; Fig. 7D EPSC10/ESPC1). A summary of these ratios for each stimulus pulse (EPSCn/EPSC1) for a 1 Hz train for both WT and Math 5^−/−^ is shown in Fig. 7C. For both groups, paired pulse ratios exhibited a progressive decline after the initial response that stabilized by the 5th pulse (two-way ANOVAs with repeated measures, F_stimulus (1.415, 167.0)_ = 637.8, *p* < 0.0001). However, the magnitude of depression was significantly greater during the second postnatal week (red circles) than either the third (green squares) or fourth (blue triangles) weeks (two-way ANOVAs, WT: F_age(2,79)_ = 13.63, *p* < 0.0001, Math 5^−/−^: F_age(2,39)_ = 11.98, *p* < 0.0001; Tukey’s multiple comparison tests: week 2 vs. week 3 or 4: *p* < 0.0001 in both WT and Math 5^−/−^).

To quantify the degree of synaptic depression at different temporal frequencies (0.25, 0.50, 1, 5, 10, and 50 Hz) we calculated the paired pulse ratios between the 10th and 1st response (Fig. 7D). For both groups, there was an age dependent depression with higher temporal frequencies exhibiting the greatest decrements (two-way ANOVAs with Tukey’s multiple comparison testing, WT: F_week(2,410)_ = 6.131 *p* = 0.0024, F_frequency(5,410)_ = 92.47, *p* < 0.0001; Math 5^−/−^: F_week(2,208)_ = 21.26, *p* < 0.0001, F_frequency(5,208)_ = 115.1, *p* < 0.0001, for both genotypes, 0.25–0.5 Hz vs. all other stimulation rates: *p* < 0.0001, 10 Hz vs. 50 Hz WT: *p* = 0.1822, Math 5^−/−^: *p* = 0.2735). Additionally, we noted that the degree of synaptic depression weakened with age with weeks 3 and 4 exhibiting larger paired pulse ratios than week 2 (WT: week 2 vs. week 3: *p* = 0.0015, week 2 vs. week 4: *p* = 0.0449, week 3 vs. week 4: *p* = 0.8169; Math 5^−/−^: week 2 vs. week 3 or 4: *p* < 0.0001, week 3 vs. week 4: *p* = 0.4586).

### Development of feedback synaptic connections between TRN and dLGN

To examine the emergence of feedback inhibitory activity from TRN to dLGN, we conducted whole cell recordings from SST-Cre x Ai32 (ChR2-EYFP) mice and ones crossed on a Math 5^−/−^ background. To determine if ChR2 expression in TRN neurons was sufficient to drive postsynaptic activity in dLGN at early postnatal ages, we recorded light evoked activity from vTRN neurons (supplemental Fig. 1C). As early as P2, ChR2-EYFP was evident in TRN and photoactivation with brief pulses (1ms) of blue light led to reliable depolarizations and spike firing (supplemental Fig. 1D).

To investigate TRN-mediated inhibition of TC neurons in dLGN, we conducted voltage clamp recordings using a cesium-based internal solution while holding neurons at 0 mV [42, 73]. We recorded 352 WT and 195 Math 5^−/−^ neurons in dLGN between postnatal ages P2-P35.

**Fig. 8:**
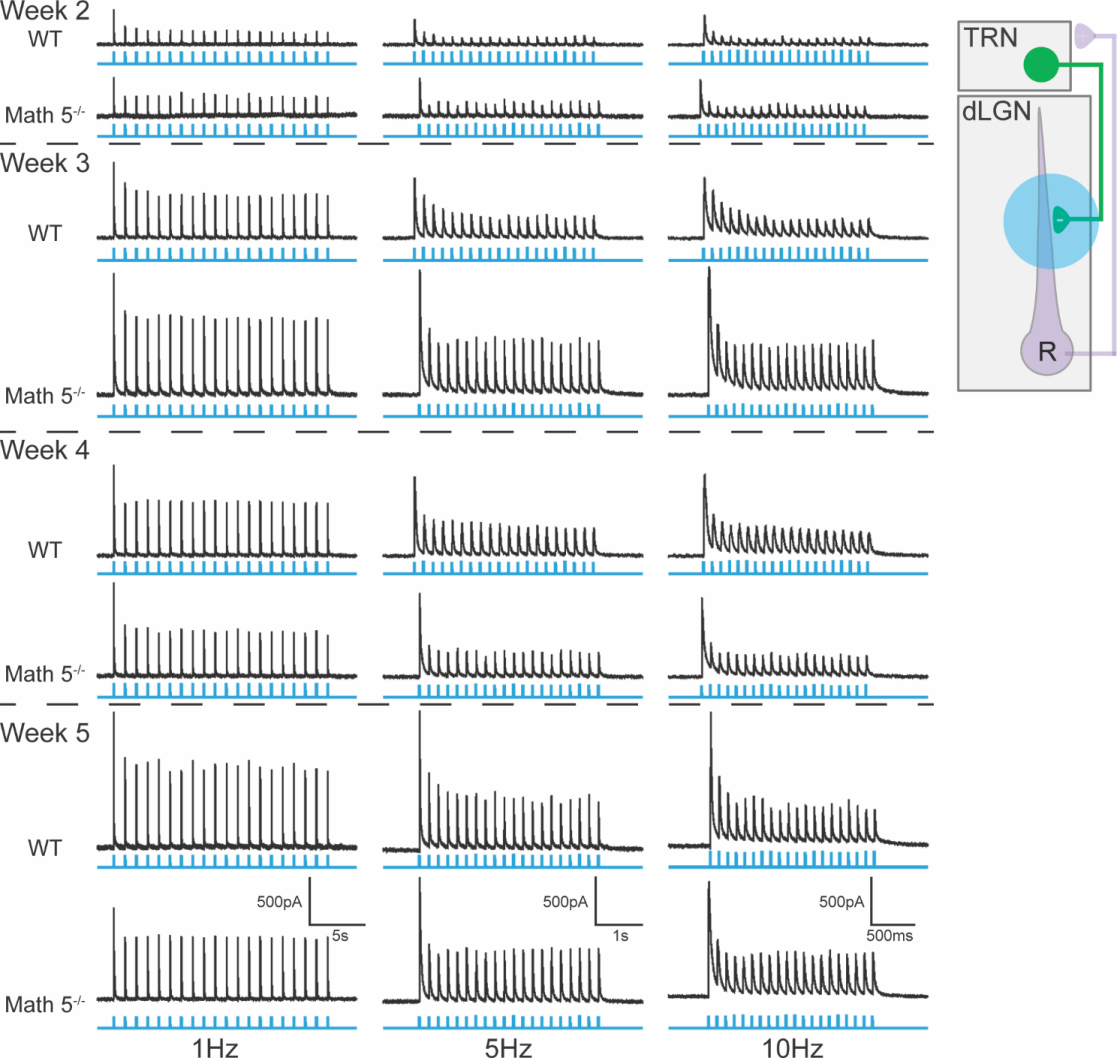
Light evoked synaptic responses of dLGN neurons from WT and Math 5^−/−^ CRH-Cre x Ai32 (ChR2-EYFP) mice. Examples of inhibitory synaptic responses recorded in voltage clamp mode to trains of blue light (twenty 1ms pulses delivered at 1, 5 and 10 Hz) are depicted by postnatal week for WT and Math 5^−/−^ mice. Trains of blue light are shown in blue beneath each corresponding response. Inset depicts feedforward (purple) and feedback green circuits between TRN and dLGN and the blue circle indicates the location of blue light stimulation. All neurons held at 0mV

Examples of the light evoked responses at different postnatal weeks to trains of blue light pulses (1ms) presented at different temporal frequencies (0.5 Hz, 5 Hz, and 10 Hz) are shown in Fig. 8. In both WT and Math 5^−/−^ dLGN, during the first postnatal week, light evoked inhibitory activity was weak and infrequent. When present, it was typically limited to the first pulse of a stimulus train. By the second postnatal week, inhibitory activity was stronger and more prevalent, but responses to repetitive stimulus trains were limited to low rates of stimulation (e.g., 0.5 Hz, 1 Hz and 5 Hz). By the start of the third postnatal week, all TC neurons exhibited light evoked inhibition that was also accompanied by a form of synaptic depression. At temporal frequencies *≥* 5 Hz, IPSC activity began to diminish after the initial pulse but stabilized midway through the stimulus train to values that were about half the amplitude of the first response.

**Fig. 9:**
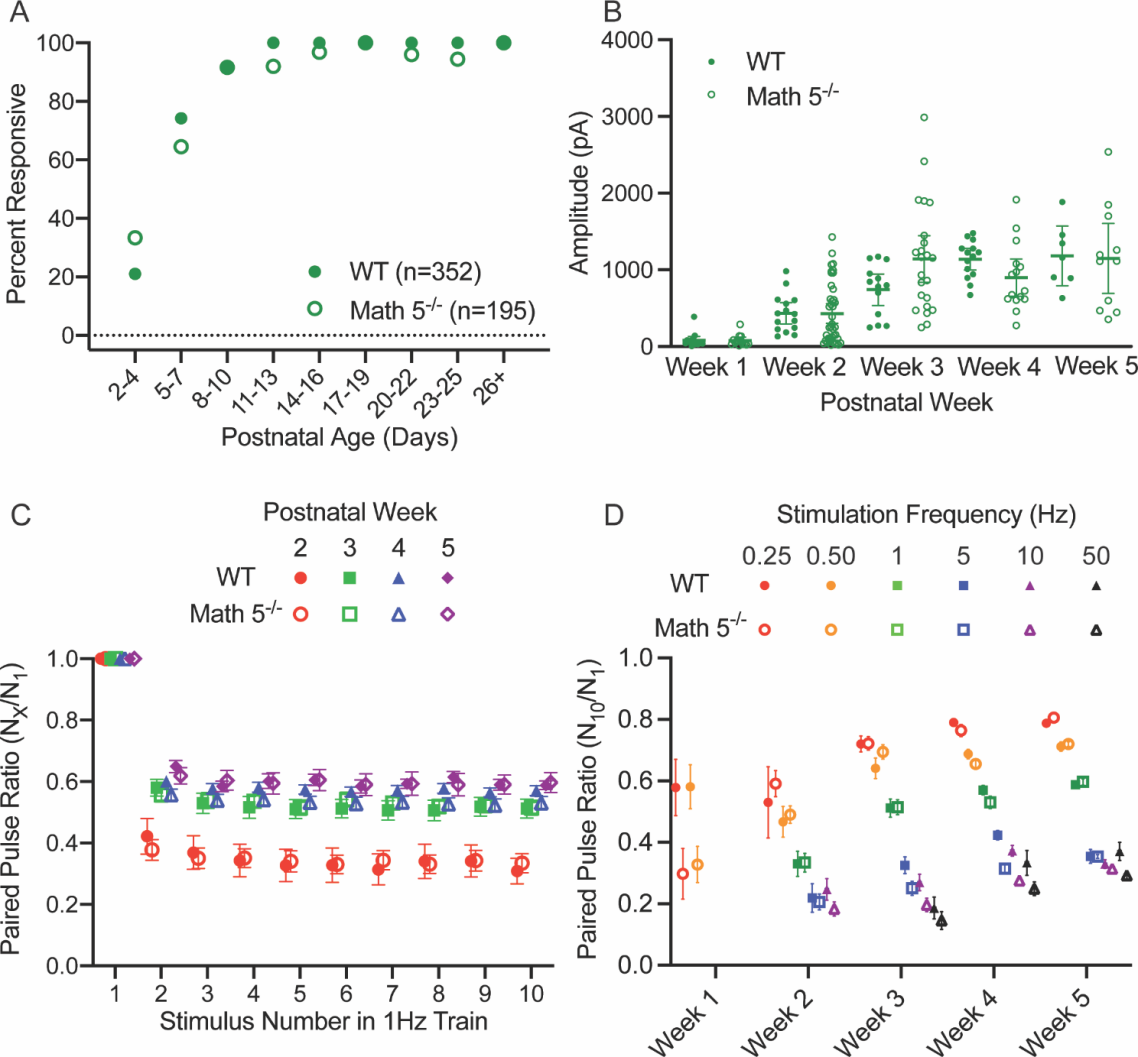
Summary of the light evoked synaptic responses of dLGN neurons from WT and Math 5^−/−^ CRH-Cre x Ai32 (ChR2-EYFP) mice. (**A**) A plot of the percent of responsive cells by age for WT (solid symbols, *n* = 352) and Math 5^−/−^ (open symbols, *n* = 195) mice. Each point represents data from 7–66 neurons (median = 25). For both groups incidence increased with age, nearing 100% by P8. (**B**) The amplitude of the initial (maximal) EPSC is plotted by postnatal week in WT (solid symbols, *n* = 64) and Math 5^−/−^ (open symbols, *n* = 102). (**C**) A plot of the paired pulse ratios as a function of stimulus number for a 1 Hz stimulus train. Ratios of EPSC amplitudes were calculated by dividing the n^th^ response by the initial response. Each point represents the mean (± SEM) of 7–15 neurons. PPRs < 1 reflect synaptic depression. Symbols depict different weeks with solid ones representing WT and open ones Math 5^−/−^ groups. Both groups showed comparable degrees of depression with the largest attenuation noted during week 2. (**D**) A plot of the paired pulse ratios as a function of age for 0.25, 0.50, 1, 5, 10 and 50 Hz stimulus trains. Ratios of EPSC amplitudes were calculated by dividing the 10th response by the initial response. Each point represents the mean (± SEM) of at least 4–40 neurons (median = 13). Temporal frequency of stimulation (0.25–50 Hz) is organized by color. Stimulus frequency led to low PPRs, which indicates a greater degree of synaptic depression

A summary of these age-related inhibitory responses for WT and Math 5^−/−^ mice are depicted in Fig. 9 where we plot the incidence of light evoked responses, (Fig. 9A), the amplitude of the initial evoked (and maximal) IPSC (Fig. 9B), and paired pulse ratios (Fig. 9C EPSCn/EPSC1; Fig. 9D EPSC10/ESPC1). For both groups, the incidence of light evoked responses followed a similar time course (Fisher’s exact test, *p* > 0.05 at all ages) with IPSC activity emerging as early as P4 (P4 WT: 5/14, 36%, Math 5^−/−^: 33%) and increasing rapidly thereafter so that by the end of the first postnatal week virtually all TC neurons exhibited responses (P7 WT: 18/21, 86%; Math 5^−/−^: 8/9, 89%, P10-12 WT: 18/18, 100%, Math 5^−/−^: 11/11, 100%; > P12 WT: 198/198, 100%, Math 5^−/−^: 116/121, 96%). Both groups showed a comparable age-related increase in IPSC amplitude (Fig. 9B; two-way ANOVA with Tukey multiple comparison testing, F_age(4,156)_ = 30.26, *p* < 0.0001; F_genotype(1,156)_ = 0.1095, *p* = 0.7412; ). Between weeks 1–3, IPSC amplitude exhibited a progressive increase (WT or Math 5^−/−^: week 1 vs. week 3, 4 or 5: *p* < 0.01, week 2 vs. week 4 or 5: *p* < 0.05) that stabilized after week 3 (WT or Math 5^−/−^: week 3 vs. week 4 or 5: *p* > 0.05).

For both groups, measurements of paired pulse ratios taken for a 1 Hz stimulus train (Fig. 9C EPSC_n_/EPSC_1_) or across a range of temporal frequencies (0.25, 0.50, 1, 5, 10 and 50 Hz; Fig. 9D) reflected a sustained synaptic depression. At 1 Hz, ratios computed for each pulse within the stimulus train show an initial reduction that remained constant throughout stimulation with the greatest attenuation seen prior to the third postnatal week (Fig. 9C; two-way ANOVA with repeated measures, F_age(7,115)_ = 11.31, *p* < 0.0001). However there was no difference between WT (solid symbols) and Math 5^−/−^ (open symbols; two-way ANOVAs, week 2: F_genotype (1,41)_ = 0.0003, *p* = 0.9866; week3: F_genotype (1,32)_ = 0.0620, *p* = 0.8049; week 4: F_genotype (1,26)_ = 2.911, *p* = 0.0999; week 5: F_genotype (1,16)_ = 0.0048, *p* = 0.9455). When examined across a range of temporal frequencies, paired pulse ratios based on the 10th pulse revealed a sustained depression, with higher temporal frequencies exhibiting the largest decline (two-way ANOVAs WT: F_frequency(5,231)_ = 60.54, *p* < 0.0001; Math 5^−/−^: F_frequency(5,385)_ = 187.0, *p* < 0.0001; Tukey multiple comparison testing in both WT and Math 5^−/−^: 0.25 Hz, 0.5–1 Hz vs. 5 Hz, 10–50 Hz: *p* < 0.0005). Such depression weakened with age (two-way ANOVAs WT: F_week(4,231)_ = 24.03, *p* < 0.0001; Math 5^−/−^: F_week(4, 385)_ = 61.23, *p* < 0.0001; within each stimulus and genotype, week 1 or week 2 vs. weeks 3–5: *p* < 0.0001, week 4 vs. week 5: *p* > 0.05) but still remained robust for both groups.

Finally, we assessed how the emergence of feedback inhibition from TRN influenced spike activity of dLGN TC neurons. We conducted current clamp recordings (WT *n* = 52, Math 5^−/−^*n* = 47 cells) and photoactivated TRN terminals while injecting a depolarizing current pulse to trigger a steady train spiking in TC neurons. Changes in spike firing were then calculated by comparing equivalent periods (0.5 s) of activity in the presence or absence of blue light stimulation (10–50 Hz).

**Fig. 10:**
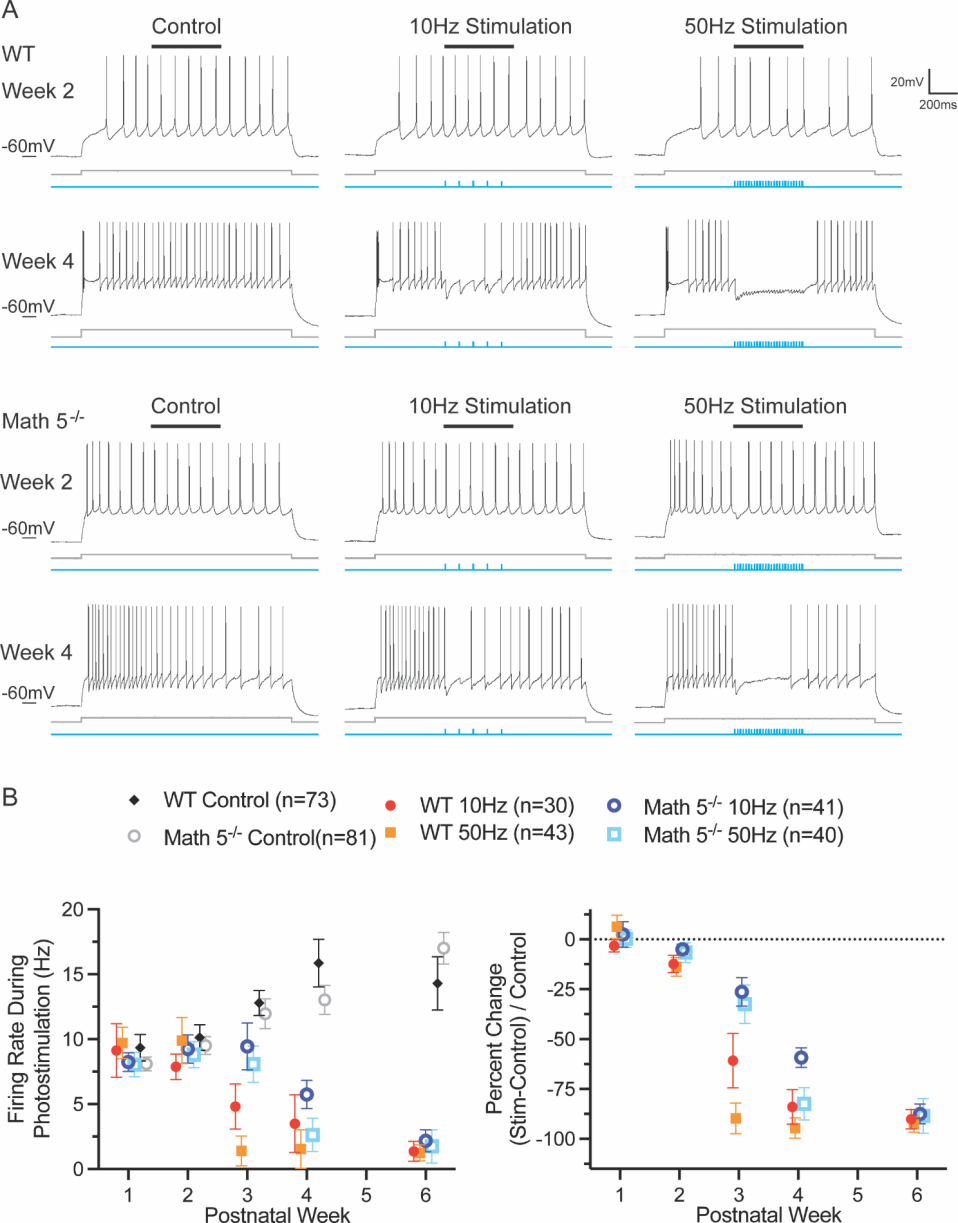
Development of TRN-mediated inhibition of dLGN activity. (**A**) Examples of voltage responses obtained from P14 (week 2, top) and P28 (week 4, bottom) SST-Cre x Ai32 (ChR2-EYFP) WT and SST-Cre x Ai32 mice crossed on a Math 5^−/−^ background. Each group shows spiking evoked by a square wave current pulse (1500ms) injection (gray trace beneath response). Responses in the left column show spiking in the absence of TRN stimulation (control), while those on the right depict responses during blue light stimulation at 10 Hz and 50 Hz. The rate of firing was measured during the middle 500ms of the depolarization (black bar, top) in the absence (control, left) and presence (stimulation, right) of blue light pulses (blue). (**B**) Summary plots showing the changes in spike firing brought about by TRN stimulation. Left: Summary of firing rates measured as a function of postnatal week during a 500ms (see above) epoch in the absence (control) and presence of TRN terminal stimulation. Each point represents the mean ± SEM for WT (solid symbols, *n* = 73) and Math 5^−/−^, (open symbols, *n* = 81) neurons. Firing rates during control recordings remained relatively stable. TRN activation had little impact on firing during the first two postnatal weeks but suppressed firing during weeks 4 and 6. Right: Plot showing firing rate as a percent change from a matching period of control. Each point represents the mean ± SEM for WT and Math 5^−/−^ groups. There was a progressive decline after week 2

Examples for WT (top) and Math 5^−/−^ (bottom) neurons recorded at 2 and 4 weeks are shown in Fig. 10A in which a square current pulse (gray, 1500ms) evoked tonic firing before (left) and during blue light stimulation (500ms, blue) at 10 Hz (middle) and 50 Hz (right; see also supplemental Fig. 2 for recordings at week 1–4, and 6). For both groups, during postnatal weeks 1 and 2, activation of TRN terminals at 10–50 Hz had little impact on spike firing. However, by week 3, TRN activation began to reduce TC spiking, and by week 4, it led to an almost complete suppression of activity. These data are summarized in Fig. 10B which plots the average firing rate (left) observed during control and photostimulation epochs. In the absence of photostimulation, both WT and Math 5^−/−^ groups showed comparable firing rates (three-way ANOVA, mixed effects analysis of control epoch: F_week(4,90)_ = 7.626, *p* < 0.0001, F_genotype(1,90)_ = 0.06348, *p* = 0.8017). During photostimulation, there was an age-related suppression of TC spiking that emerged at week 3, continued to decline through week 4 and then led to a nearly complete suppression by week 6 (three-way ANOVA, mixed effects analysis: F_week(4,90)_ = 13.73, *p* < 0.0001; Tukey multiple comparison testing: week 1 or 2 vs. week 4 or 6: *p* < 0.0005). Further post hoc testing revealed that a significant difference in spiking between WT and Math5-/- at week 3, with neurons from WT showing lower values than Math 5-/- (Tukey multiple comparisons: week 3 WT vs. Math5-/- 10 Hz *p* = 0.049, 50 Hz *p* = 0.0159).

A similar pattern emerged when firing rates are converted to percent change (stim-control/control) in activity (Fig. 10B, right) with values approaching 100% reduction at 6 weeks of age (mean +/- SEM: WT 10 Hz: 90.107 +/- 4.893, 50 Hz: 92.755 +/- 3.916, Math 5^−/−^ 10 Hz: 87.517 +/- 5.035, 50 Hz: 88.483 +/- 8.670).

## Discussion

These studies provide new information about the postnatal development of the feedforward and feedback projections between dLGN and TRN. Our tracing experiments reveal that thalamocortical and perhaps even anterogradely labeled corticothalamic, axons passed through TRN in an orderly, modality-specific manner that resembles the arrangement noted in adult mice. As early as P2 there was a well-defined and non-overlapping organization of sensory afferents, with visual TC axons restricted to a region that is dorsal to the apex and in the head of TRN while nonvisual ones pass through the “tail”, ventral to the apex [44, 52, 74]. Moreover this sectorial arrangement persists even in the absence of retinal innervation (see also [75, 76]), suggesting that the organization of TRN is determined largely by intrinsic guidance cues and seems impervious to alterations in signaling from the periphery [62, 77].

**Fig. 11:**
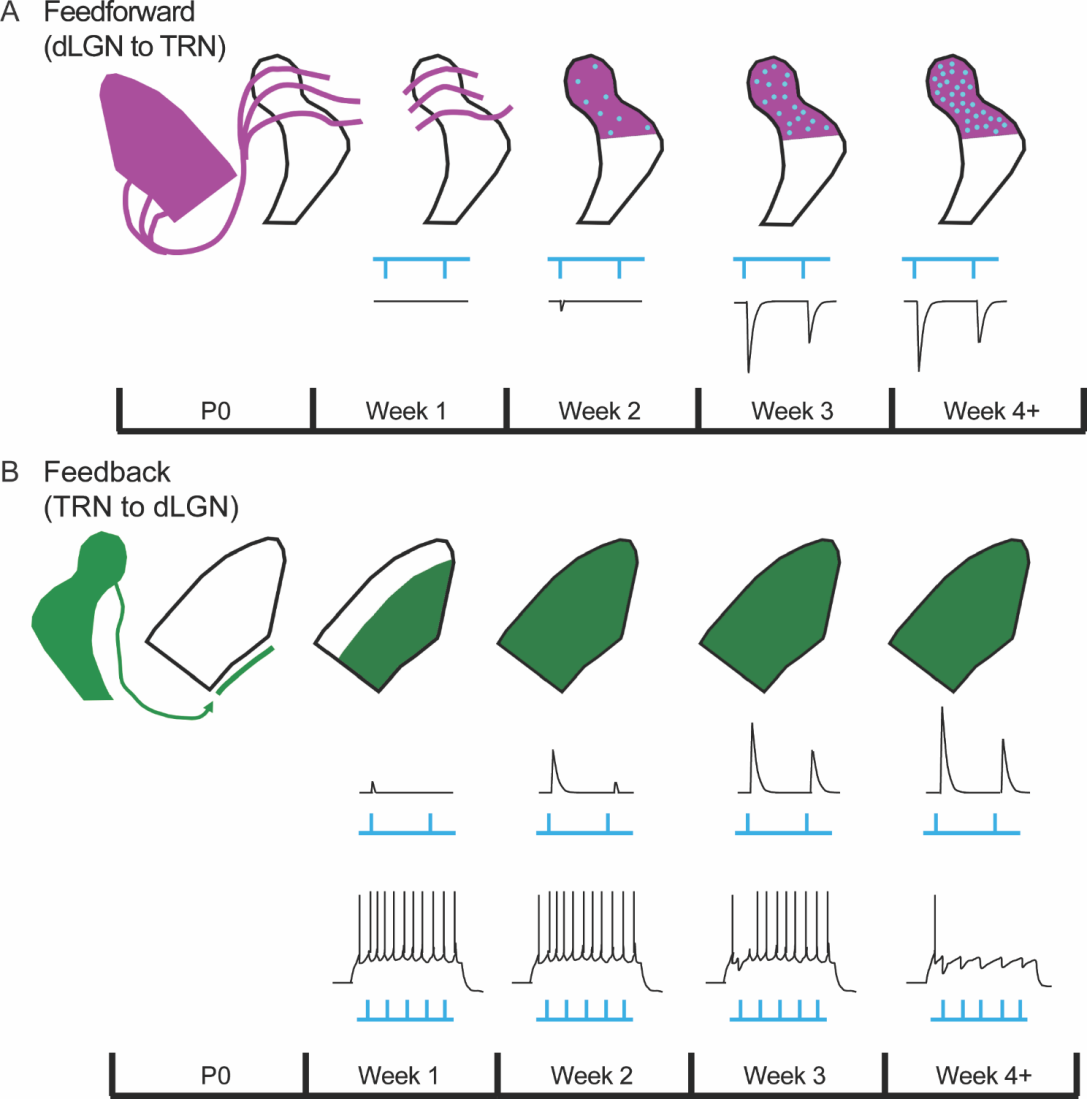
Timeline illustrating the development of the connections between TRN and dLGN. (**A**) Development of feedforward input from dLGN to TRN. Top: Thalamocortical projections (purple) are present in TRN at birth but terminals on axon collaterals (blue) do not emerge until postnatal week 2. The density of axon collateral terminals gradually increases up until postnatal week 4. Bottom: Excitatory responses are absent during the first postnatal week and highly immature during week 2. EPSCs are weak and exhibit fatigue in the face of repetitive stimulation. EPSC activity gradually strengthens so that by week 3, evoked activity is of large amplitude and responds faithfully to repetitive stimulation. (**B**) Development of TRN feedback projections to dLGN. Top: TRN fibers (green) are present at the ventral-medial edge of dLGN at birth. During week 1, fibers innervate dLGN and cover much of the nucleus. Middle: Inhibitory responses arise during postnatal week 1 but are of low amplitude and cannot follow trains of stimuli > 0.5 Hz. Responses continue to mature during postnatal weeks 2 and 3 so that by week 4, evoked activity is large and able to follow high temporal frequency stimulation. Bottom: TRN inhibition has little if any impact on dLGN spiking during the first two postnatal weeks. During week 3, activation of TRN terminals decreases TC firing, and by week 4 can fully suppress transmission

Our anatomical and electrophysiological experiments in strains that isolate the reciprocal connections between TRN and dLGN revealed that feedback connections from TRN to dLGN were established before feedforward TC collateral input onto TRN neurons. (Fig. 11). TRN axons passed through the ventral-medial border of dLGN at birth and began to innervate dLGN at P2 (Fig. 11, green), fully encompassing the nucleus by P7. At this time, optogenetic stimulation of TRN terminals in dLGN led to weak, light-evoked inhibitory postsynaptic activity among TC neurons. Over the course of the second and third postnatal weeks, responses increased in amplitude and showed some form of synaptic suppression during repetitive stimulation. During weeks 3–5 this depression remained relatively strong, especially during high rates of repetitive stimulation. Interestingly, TRN mediated inhibition had little impact on TC firing during weeks 1–2, perhaps because synaptic currents remained relatively weak. However, the continued age-related increase in current strength between weeks 3–5 led to a concomitant reduction in TC spiking. Indeed, by week 4 TRN stimulation led to an almost complete suppression of TC spiking, especially at high rates of stimulation [36]. The synaptic depression noted during weeks 1–2 may in part reflect the immature state of a developing synapse since even low rates of stimulation evoked responses that showed substantial fatigue. However, the depression noted in subsequent weeks is likely to reflect the emergence of an adult-like property that is consistent with their ultrastructural synaptic profile [36].

By contrast, excitatory feedforward connections between TC collaterals and TRN neurons appear later. Although TC axons passed through visual TRN at birth, the labeling of axon collaterals with vGluT2 was not detected until the second week (P10) and did not fully encompass visual TRN until the third week. The onset of synaptic activity followed a similar trajectory. Optogenetic stimulation of TC collaterals in TRN evoked weak excitatory responses during the second week which peaked in amplitude by the end of the third week. Accompanying this increase in synaptic strength was a frequency dependent depression, suggesting that excitatory TC input to TRN is driver-like in nature [78, 79].

While retinal afferents are the first to arrive to dLGN and make functional connections with TC neurons [80–82], TRN feedback connections seem to precede the arrival and establishment of other nonretinal inputs [24]. For example, descending CT projections from layer 6 begin to arrive in force and make connections with TC neurons during the second postnatal week [29, 83], while those from brainstem cholinergic nuclei take weeks to arrive and fully innervate dLGN [44]. Indeed, feedback inhibition from TRN to dLGN also occurs prior to the onset of feedforward inhibition from GABAergic intrinsic interneurons [42], which form connections with TC neurons near the end of the second week [81]. The latter sequence has important implications for understanding the source of thalamic inhibition as a contributing factor underlying the maturation of TC network dynamics. The emergence of thalamic inhibition has been postulated to account for the transformation of network activity from an immature state dominated by weak intrinsic oscillatory activity to a more mature one that is comprised of stimulus evoked events and sleep/wake EEG patterns [21–23]. The early postnatal arrival of TRN feedback inhibition appears to roughly coincide with the maturation of TC network dynamics and thus makes for a suitable candidate mechanism.

Finally, it is important to note the role of retinal innervation in intrathalamic circuit formation. Our results in Math5^−/−^ mice, a mutant that is devoid of retinofugal projections [31, 33], indicate that the loss of retinal input accelerates TRN innervation of dLGN, but has little or no impact on the development of dLGN input to TRN. The premature innervation of dLGN by TRN afferents is consistent with several other studies that underscore the role of retinal signals in regulating the timing of non-retinal innervation of dLGN [24]. Indeed, in the absence of retinal innervation, intrinsic interneurons fail to target dLGN appropriately, leading to a loss of their input onto TC neurons [42, 84]. It also results in a delay and misrouting of brainstem cholinergic input to dLGN [44], as well as an acceleration of descending layer VI innervation of dLGN [29]. The latter is mediated by the early degradation of aggrecan an extracellular matrix molecule that serves as a repellent for CT innervation of dLGN [28]. However, in the absence of retinal input this repulsive molecule is nearly absent at perinatal ages due to abnormally high levels of degradative proteases that cleave it. Whether aggrecan also regulates the timing of TRN innervation remains untested. Interestingly, the absence of retinal input to dLGN did not appear to alter the timing or nature of TRN synapse formation. The incidence, strength, and degree of synaptic suppression were unaffected. Therefore, the presence of retinal inputs at early ages, while altering the rate of TRN innervation, does not appear to have an impact on TRN synapse formation or maturation. This would suggest that these developmental steps in circuit assembly (innervation and synapse formation) are separable and regulated by independent mechanisms.

## Conclusion

The TRN is organized into modality specific sectors at or near birth. The feedback connections from TRN to dLGN are established before feedforward TC collateral input onto TRN neurons. While TC axons pass through TRN at birth, their collateral sprouting and synaptic innervation of TRN neurons occurs largely during weeks 2–3. Feedback projections from TRN to dLGN begins at P2, with terminals encompassing most of dLGN by the end of the first week and functional inhibitory responses emerging soon after, reaching an adult-like state by 3 weeks of age. There is an age-related increase in TRN mediated inhibition which is accompanied by a progressive increase in the suppression of TC spiking. The absence of retinal innervation leads largely to an acceleration of TRN innervation of dLGN but has little impact on the development of feedforward projections from dLGN to TRN. Together, these studies provide a foundation to investigate the development of TC network dynamics as well as a reference to understand neurodevelopmental diseases that implicate TRN circuitry.

### Electronic supplementary material

Below is the link to the electronic supplementary material.

Supplementary Material 1: ChR2 expression in developing feedforward and feedback thalamic circuits. **A.** Left: wiring diagram illustrating somatic blue light stimulation of thalamocortical (TC) neurons comprising the feedforward circuit to TRN. Right: Examples of intrinsic ChR2-EYFP expression in TC neurons and their projections in P5 CRH-Cre x Ai32 mice. There was robust EYFP in TC neurons of dLGN (outlined) ventrobasal complex (VB) as well as in their projections coursing through TRN (outlined) and terminating in a barrel like arrangement of the somatosensory cortex. Sections were cut in the coronal plane. **B**. Left: Coronal slice through the thalamus of a P4 CRH-Cre x Ai32 mice showing ChR2-EYFP expression in dLGN and VB along with the location of biocytin filled TC neurons (white). Middle: Z-stack confocal images of biocytin filled TC neurons from adjacent slice. Right: Voltage responses and spikes evoked by blue light stimulation (blue trace, 1ms pulse) of TC neurons. **C**. Left: wiring diagram illustrating somatic blue light stimulation of TRN neurons comprising feedback circuit to dLGN. Right: Examples of intrinsic ChR2-EYFP expression in TRN neurons and their projections to dLGN at P2 and P6 of SST-Cre x Ai32 mice. There was robust EYFP in TRN neurons (outlined) as well as their terminal fields in dLGN (outlined). Sections were cut in the coronal plane. **D**. Left: Coronal slice through the thalamus of a P2 SST-Cre x Ai32 mice showing ChR2-EYFP expression in TRN along with the location of biocytin filled neurons (white). Middle: Z-stack confocal images of biocytin filled TRN neurons from adjacent slice. Right: Voltages responses and spikes evoked by blue light stimulation (blue trace, 1ms pulse) of TRN neuron

Supplementary Material 2: Development of TRN-mediated inhibition of dLGN activity. Examples of voltage responses obtained from SST-Cre x Ai32 (ChR2-EYFP) WT (top) and SST-Cre x Ai32 mice crossed on a Math5^−/−^ background (bottom). Responses are organized by postnatal week and stimulation frequency (10 Hz and 50 Hz). All other conventions same as Fig. 10A

## Data Availability

The datasets used and/or analyzed during the current study are available from the corresponding author on reasonable request.
