# Supplementary figures and images for: Development of reciprocal connections between the dorsal lateral geniculate nucleus and the thalamic reticular nucleus
Source: Neural Dev. 2024 Jun 18;19:6. doi: 10.1186/s13064-024-00183-5 (PMC11184795; doi:10.1186/s13064-024-00183-5)

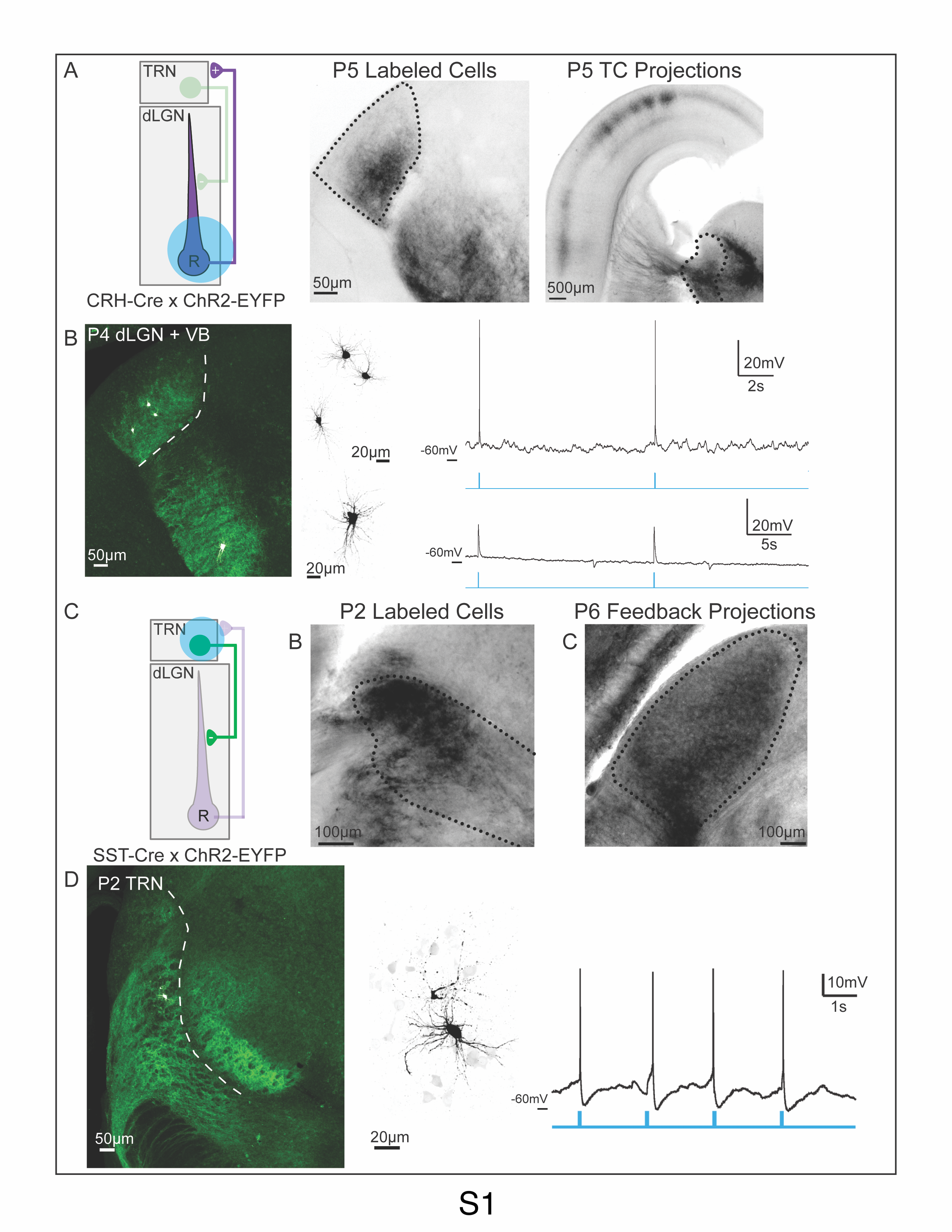

Supplement: Supplementary file 1 — Supplementary Material 1: ChR2 expression in developing feedforward and feedback thalamic circuits. A. Left: wiring diagram illustrating somatic blue light stimulation of thalamocortical (TC) neurons comprising the feedforward circuit to TRN. Right: Examples of intrinsic ChR2-EYFP expression in TC neurons and their projections in P5 CRH-Cre x Ai32 mice. There was robust EYFP in TC neurons of dLGN (outlined) ventrobasal complex (VB) as well as in their projections coursing through TRN (outlined) and terminating in a barrel like arrangement of the somatosensory cortex. Sections were cut in the coronal plane. B. Left: Coronal slice through the thalamus of a P4 CRH-Cre x Ai32 mice showing ChR2-EYFP expression in dLGN and VB along with the location of biocytin filled TC neurons (white). Middle: Z-stack confocal images of biocytin filled TC neurons from adjacent slice. Right: Voltage responses and spikes evoked by blue light stimulation (blue trace, 1ms pulse) of TC neurons. C. Left: wiring diagram illustrating somatic blue light stimulation of TRN neurons comprising feedback circuit to dLGN. Right: Examples of intrinsic ChR2-EYFP expression in TRN neurons and their projections to dLGN at P2 and P6 of SST-Cre x Ai32 mice. There was robust EYFP in TRN neurons (outlined) as well as their terminal fields in dLGN (outlined). Sections were cut in the coronal plane. D. Left: Coronal slice through the thalamus of a P2 SST-Cre x Ai32 mice showing ChR2-EYFP expression in TRN along with the location of biocytin filled neurons (white). Middle: Z-stack confocal images of biocytin filled TRN neurons from adjacent slice. Right: Voltages responses and spikes evoked by blue light stimulation (blue trace, 1ms pulse) of TRN neuron [file 13064_2024_183_MOESM1_ESM.tif]

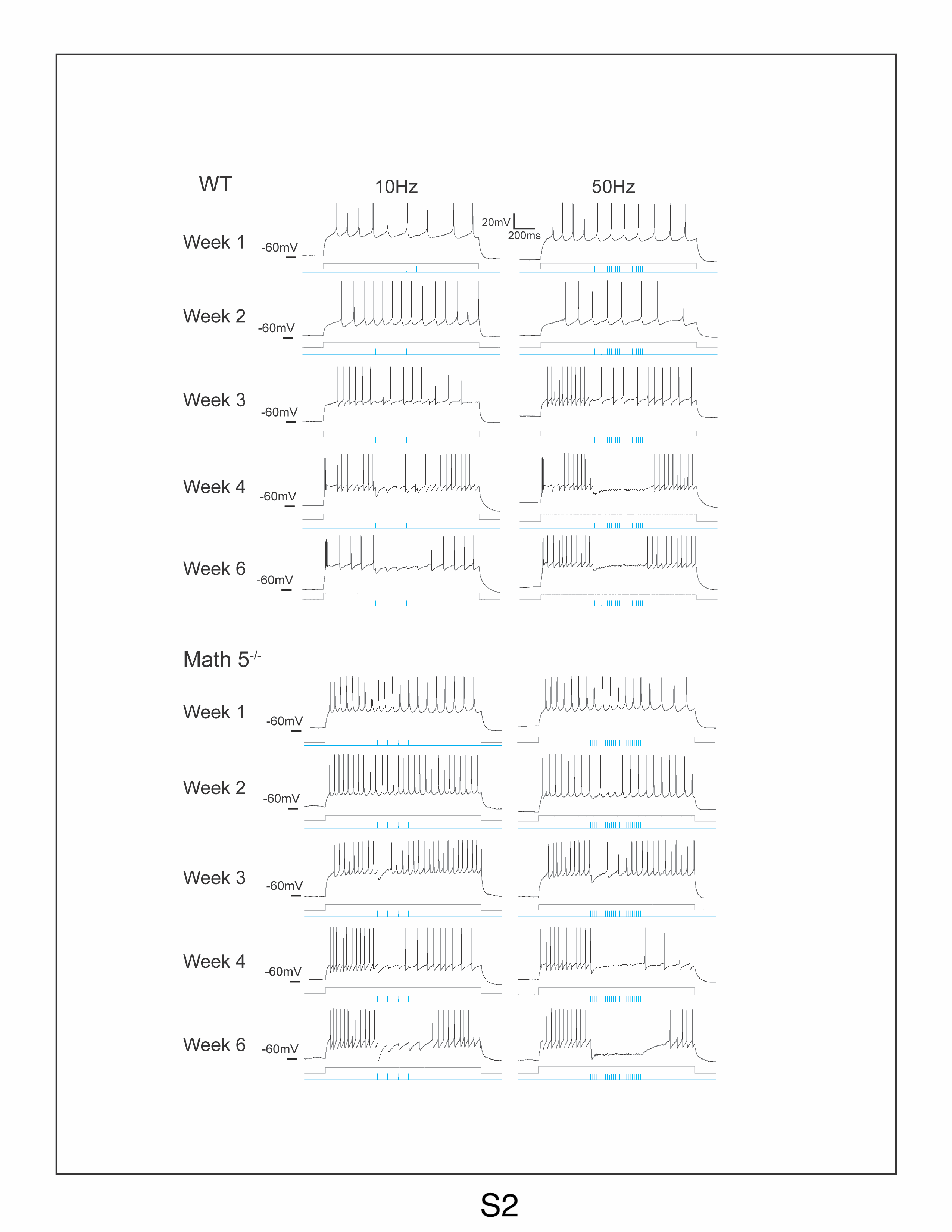

Supplement: Supplementary file 2 — Supplementary Material 2: Development of TRN-mediated inhibition of dLGN activity. Examples of voltage responses obtained from SST-Cre x Ai32 (ChR2-EYFP) WT (top) and SST-Cre x Ai32 mice crossed on a Math5−/− background (bottom). Responses are organized by postnatal week and stimulation frequency (10 Hz and 50 Hz). All other conventions same as Fig. 10A [file 13064_2024_183_MOESM2_ESM.tif]
